# A systematic review of task-switching in healthy older adults: paradigm manipulations and switch costs

**DOI:** 10.1007/s00426-026-02398-z

**Published:** 2026-11-12

**Authors:** Xuanning He, Erin I. Walsh, Anne M. Aimola Davies

**Affiliations:** The Australian National University, Building 39, Science Rd, Canberra, 2601 ACT Australia

## Abstract

It is widely accepted that task-switching performance changes in later-life, but it is currently unclear how differences in measurement accrued over almost one hundred years may be affecting our understanding of task-switching performance differences in young *versus* older adults. This review investigated the different manipulations used in task-switching paradigms with healthy older adults (age 59–97 years), and the impact of these differences on task-switching performance. A search across four databases resulted in 73 articles meeting the inclusion criteria. We found considerable variability in how task-switching in later-life was operationalised, with increasing complexity in paradigm design over time. Digit tasks were the most common task-switching task, and word-cued switching was the most common switch instruction. Larger switch costs were correlated with complex stimulus features, task environments with less informative cues, and shorter preparation time between trials. Most studies comparing young and older adults have found that task-switching was slower and less accurate in older adults. However, conclusions were limited by substantial under-reporting of non-significant results. Future research with better consistency in reporting is needed to clarify whether paradigm manipulations impact the fundamental goal of accurately capturing the costs of switching when using task-switching paradigms in healthy young and older adults.

**Supplementary Information:**

The online version contains supplementary material available at https://doi.org/10.1007/s00426-026-02398-z.

## Highlights

- Task-switching paradigms have increasingly varied over time.
- Task-switching paradigm design effects the magnitude of task-switching performance.
- Reporting of demographic and statistical information varied substantially.
- The tasks and switch methods in task-switching paradigms are important factors.

**Supplementary Information:**

The online version contains supplementary material available at https://doi.org/10.1007/s00426-026-02398-z.

## Introduction

Task-switching, also known as set-shifting or cognitive-flexibility, is the ability to alternate sequentially between multiple tasks (Ging-Jehli & Ratcliff, 2020; Jersild, 1927). To switch tasks successfully, executive control processes are needed to coordinate the inhibition of the old task with the activation of the new task (Allport et al., 1994). This cognitive process has been explored in laboratory settings using tasks including the Wisconsin Card Sorting Test (Berg, 1948) and the Trail Making Test (Caselli et al., 2020), and most commonly through a task-switching paradigm built on the foundational design by Jersild (1927). The task-switching paradigm has evolved greatly since 1927, but the basic concept and procedures have remained the same. Participants are required to switch sequentially between two (or more) base-tasks. In a task-switching paradigm, switching can be implemented into different components of the tasks, such as attention, selection criterion, modality, or the skills required to complete the task. However, in the current review, we have taken the approach that different base-tasks require different skills to complete the task.

Before considering the influence of base-task choice further, it is useful to clarify what is meant by the terms task and task switching. Following Koch et al. (2018), a task can be defined broadly as a configuration of cognitive processes linking a class of stimuli to a class of responses, decomposable into several components: selection of the relevant stimulus or stimulus attribute (e.g., a particular sensory modality or perceptual dimension), the rule mapping that is attributed to a response category (i.e., the stimulus-response, or S-R, mapping), and the selection and execution of the response itself. Task-switching refers to a change in one or more of these components from one trial to the next. Paradigms differ considerably in how many, and which, components change with each switch. Some paradigms change several components simultaneously (e.g., Rogers & Monsell, 1995, in which both the relevant stimulus feature, digit *versus* letter of a compound stimulus, and the S-R mapping rule, parity *versus* vowel/consonant status, changed together), whereas other paradigms isolate a single component (e.g., Meiran, 1996, in which the same spatial judgement was required throughout and only the cued stimulus dimension, vertical *versus* horizontal position, changed; see also Schils et al., 2024). This distinction is conceptually separate from base-task choice; base-task choice concerns which broad skill domain a task draws upon (e.g., colour perception, semantic categorisation).

**Fig. 1:**
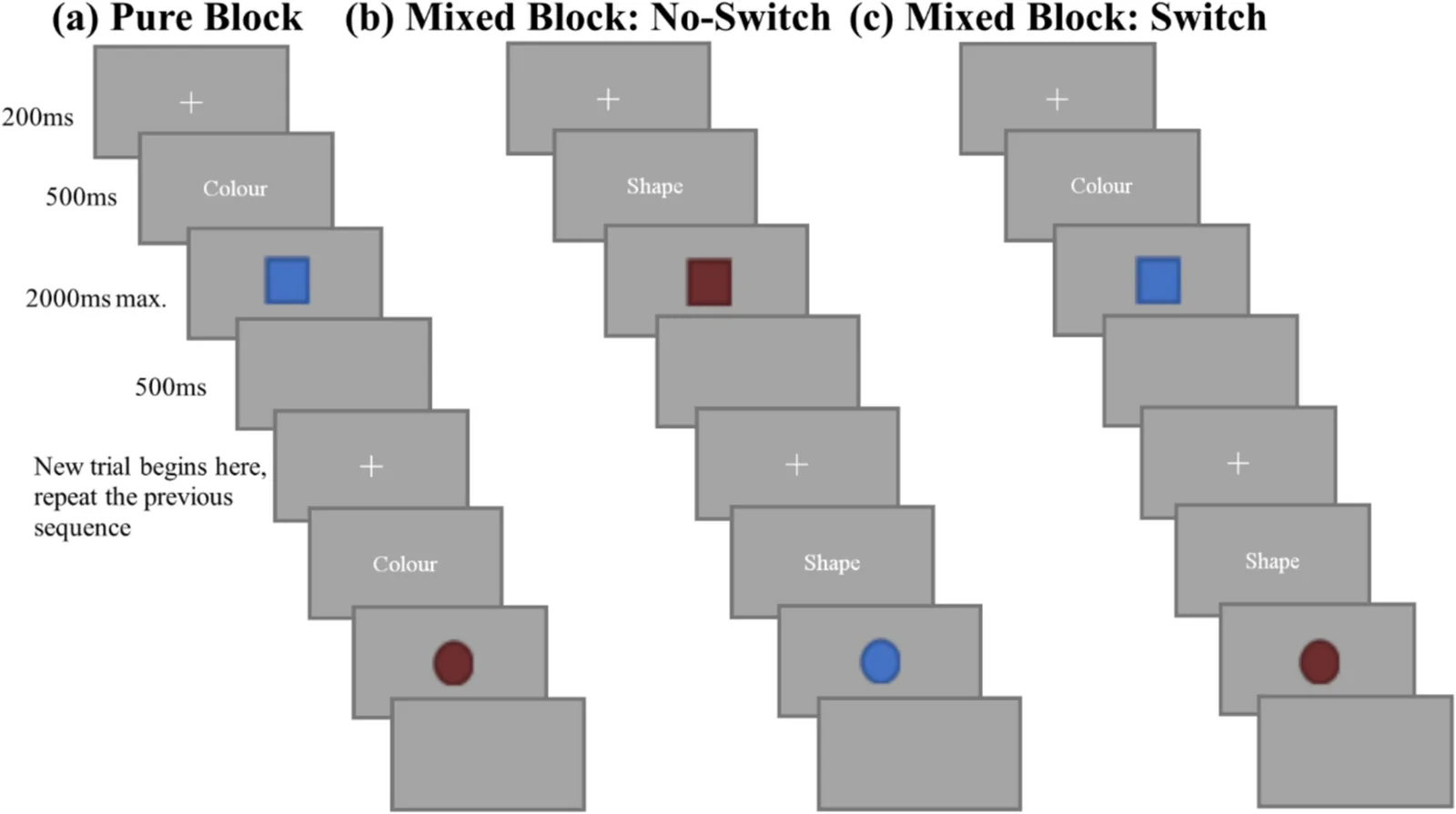
Example of a task-switching paradigm (Figure modified based on Vasta et al., 2018). Note. Typical example of a task-switching paradigm. Maximum trial length is 3200ms, starting with fixation cross (200ms) in centre of screen, followed by cue (500ms) instructing participants to perform Task-A (cue: Colour) or Task-B (cue: Shape). Next, stimulus appears, and participants make forced-choice response within 2000ms. Trial ends with blank screen (500ms), and new trial begins that repeats previous sequence, until all trials are completed. In each trial, participants perform either Task-A, colour-discrimination task (i.e., colour of the stimulus is blue or red), or Task-B, shape-discrimination task (i.e., shape of the stimulus is circle or square). In a pure block (Panel **a**), participants perform a single task throughout the entire block. Only one task-set needs to be activated at any time, and no task selection is required. In a mixed block (Panel b and Panel c), both Task A and Task B may occur on any trial. On no-switch trials within a mixed block (Panel **b**), participants perform the same task as on the immediately preceding trial, the trial-level action is therefore identical to a pure block trial. However, the block-level context differs because a switch to the other task is possible at any point, participants must prepare for both task-sets simultaneously and remain ready to select between them on every trial, regardless of whether a switch actually occurs on that trial. On switch trials within a mixed block (Panel c), participants perform the task-switching as instructed. Panel (**a**) presents an example of two trials from Task-A pure-block, in which participants perform only Task-A (cue: Colour). (Task-B pure-block is not presented in this figure but in Task-B, participants would perform only Task-B (cue: Shape)). Two examples of trials from the mixed-block include: Panel (**b**) a no-switch trial, where the Task-B trial is followed by another Task-B trial, and Panel (**c**) a switch trial, where the Task-A trial is followed by a Task-B trial.

Examples of possible base-tasks have included digit-parity tasks (i.e., a choice as to whether the digit presented was an odd number or an even number), colour-discrimination tasks (i.e., a choice as to whether the colour of the stimulus presented was red or blue), and semantic-categorisation tasks (i.e., a choice as to whether the stimulus presented was a fruit or a vegetable). More than one base-task may be used. For example, in the paradigm illustrated in Fig. 1 (Vasta et al., 2017), participants were required to switch between a colour-discrimination base-task and a shape-discrimination base-task. Task-switching paradigms can also contain various stimulus features, such as the complexity of task-stimuli, the informativeness of task-cues, and the length of time between trials. Although these modifications were examined within task-switching paradigms, the specific cognitive demands and task characteristics varied across studies. Therefore, it is unclear whether differences in switch costs across paradigm manipulations reflect differences in task-switching performance itself or differences in other characteristics of the tasks and procedures.

The age-associated decline in task-switching abilities often found in later-life has been proposed to result in a reduction in the capacity of older adults to remain engaged in various tasks of daily living (Vaughan & Giovanello, 2010). As research on age-associated changes in task-switching performance is accumulating across the different variations of the task-switching paradigm, there is a need to aggregate evidence to allow for a more comprehensive understanding of ageing and task-switching. Reviews to date have either focussed on the underlying mechanisms of task-switching (e.g., as in Kiesel et al., 2010), or aggregated results across a variety of paradigm designs (e.g., as in Chen & Hsieh, 2023). We assert that it is important to understand how variability in the task-switching paradigm may impact task-switching performance, in order to unambiguously understand differences in task-switching performance attributable to age. Relevant considerations are the different measures of task-switching used in task-switching paradigms to test both young and older adults, the paradigm manipulations developed by previous researchers, sample size and effect size.

### Measuring switch costs in task-switching paradigms

Task-switching paradigms make use of two kinds of blocks: pure-blocks and mixed-blocks. In pure-blocks, participants are required to complete only one task, such as a colour-discrimination task where they respond to whether a given shape is blue or red, or a shape-discrimination task where they respond to whether a given shape is square or circle. In mixed-blocks, there is a requirement either not to switch between tasks, or to switch between tasks, such as a colour-discrimination task followed by a shape-discrimination task. Based on the information gathered from previous studies, each block in a task-switching paradigm can contain from 20 trials to hundreds of trials.

Switch costs are quantifiable measures of the cognitive processes required to make a switch between trials. Switch costs are typically measured as global-switch costs and local-switch costs, and sometimes they are referred to as mixing costs and switching costs respectively. Global-switch costs are measures of the reaction time (RT) and accuracy differences between the pure-block trials and the no-switch trials within the mixed-block of trials (i.e., no-switch trials are trials within the mixed-block that require participants to perform the same task as in the previous trial). Typically, pure-block trials are performed faster and with higher accuracy compared to no-switch trials in the mixed-blocks. Local-switch costs are measures of the RT and accuracy differences between the no-switch trials and the switch trials within the mixed-block of trials. In contrast to no-switch trials, switch trials are trials within the mixed-block that require participants to perform a different task from the previous trial. Typically, the no-switch trials of the mixed-block are performed faster and with higher accuracy compared to the switch trials of the mixed-block.

The paradigm-related factors contributing to variability in task-switching includes base-tasks, switch-rules, stimulus-features, task-environment, and preparation-time. These factors are thought to influence task-switching performance because they modulate the cognitive processes required for task-set reconfiguration and interference control (Kiesel et al., 2010). The nature of the base-task determines the cognitive demands and processing level required for completing the task, which can influence the magnitude of switch-costs. Switch-rules determine the complexity of the task-set mapping and the degree of cognitive control required to update or reconfigure the active task set. There are many other factors that go beyond the choice of base-tasks and switch-rules that can also influence performance, as measured by switch costs. When more complex stimulus-features are used in the base-tasks, they can affect the ease of identifying the features in the stimuli, resulting in longer RT and larger local-switch costs (Rey-Mermet & Meier, 2015). More complex and less informative task-environments are also associated with larger local-switch costs (Hahn et al., 2004). Longer preparation-times (i.e., a longer time between cue and target within an experimental trial) are associated with lower local-switch costs (Meiran et al., 2001). This is a non-exhaustive list. However, these are the most commonly accessed factors in the literature of task-switching in healthy older adults.

### Understanding task-switching performance in older adults through age-group comparisons

The extent to which different base-task combinations influence task-switching performance has not previously been explored. Divergence from Jersild’s (1927) original task-switching paradigm has implications for interpreting the results as they relate to task-switching performance differences between young and older adults. The pattern of age-related cognitive decline varies across different executive functions (Deary et al., 2009; McCarrey et al., 2016). Therefore, it follows that the choice of base-task is important because different base-tasks will require different cognitive processes. For instance, Vonk et al. (2020) suggested that semantic processing abilities remain intact until the age of 70–75, whereas working memory and processing speed decline from the age of 55. This is unlikely to impact young *versus* older group comparisons in task-switching paradigms that minimise working memory or processing speed, such as requiring participants to switch between a digit-discrimination task and a digit-comparison task and either indicate if a digit was odd or even, or that it was smaller or greater than five (Chang et al., 2020). However, there is likely to be an asymmetry in older adults’ task performance when engaging with a paradigm requiring participants to switch between a semantic-categorisation task and a size-comparison task, in which participants either categorised the meaning of a presented stimulus (e.g., fruit or vegetable) or compared the size of the presented stimulus to a reference, such as the computer screen (Simon & Bock, 2017). Older adults’ baseline performance on the size-comparison component would be expected to slow disproportionately relative to the semantic-categorisation component. Another source of such asymmetries comes from the phenomenon of asymmetric switch costs, where the cost of switching into a dominant or more practised task set is often greater than switching into a non-dominant one. This has been attributed to the carryover of inhibition applied to the dominant task set while the non-dominant task was being performed (Allport et al., 1994; Yeung & Monsell, 2003). If the relative dominance between two base tasks differs systematically by age, the direction and magnitude of this asymmetry could itself differ between young and older adults, rather than the age differences simply scaling uniformly across base-task pairings. As such, the category of base-task needs to be separated from participants’ task-switching performance to understand the function of task-switching independently of such confounds. Consequently, the current review investigated whether any given combination of base-task was easier to switch between than others for older adults.

Another consideration is whether different switch-rules can exert an influence on task-switching performance. Switch-rules are used to instruct participants about what kind of task-switching will be required in a particular trial, with example categories of switch-rules including switching voluntarily (e.g., Butler & Weywadt, 2013), memorising switch-rules for the whole trial block (e.g., Hakun et al., 2015), and cueing before each trial of the task (e.g., West & Moore, 2005). Switching voluntarily means that the choice to task-switch or not is made by the participant. This requires adequate planning and decision-making abilities, given that selecting which task to perform in the absence of an external instruction engages top-down control processes that are not required when switching is externally specified (Arrington & Logan, 2005; Forstmann et al., 2006). Memorising switch-rules for the whole trial block involves participants memorising in advance the switch-rules for the whole trial block. This requires intact memory abilities, as participants must hold the upcoming task sequence in mind and track their progress throughout the block (Rogers & Monsell, 1995). A common memorising switch-rule is the alternate switch-rule, also known as alternate run or predictable task-switching (Kiesel et al., 2010), in which participants switch from the previous-task to the alternate-task between each trial for the whole trial block. The alternate-switch cost is the RT and accuracy differences between the pure-block trials and the alternate-block trials, typically pure-block trials are performed faster and with higher accuracy compared to alternate-block trials. Cueing before each trial of the task is where participants either repeat or switch tasks according to the cue provided before each of the trials. Based on the review conducted by Kiesel and colleagues in 2010, the most common type of task-switching paradigm was the cued task-switching paradigm, followed by the alternate task-switching paradigm. Visual processing ability is required across all three switch-rule types in order to perceive and respond to the base-task stimuli. However, cued task-switching places an additional demand on visual processing because participants must also visually identify and interpret a cue on every trial to determine which task to perform. This is a requirement that is absent from voluntary switching where no external cue is presented, and from memorising or alternate switching where the task sequence is fixed in advance rather than signalled trial-by-trial. Hence, a component of the switch cost observed in cued task-switching paradigms has been attributed specifically to the process of encoding and identifying the cue itself, rather than to task switching (Logan & Bundesen, 2003; Mayr & Kliegl, 2003).

One possible explanation for the finding of limited age differences in some task-switching studies is the presence of external task cues. These cues may facilitate task-set retrieval and preparation processes, and consequently reduce the cognitive control demands associated with task-switching. Cue availability may attenuate age-related differences in switching performance by providing additional environmental support for task-set selection. In a recent meta-analysis, Chen and Hsieh (2023) examined whether predictable *versus* unpredictable switching moderated age differences in task-switching performance. Their analyses indicated that predictability did not significantly modulate age-related differences in local-switch costs. This finding suggests that although structural properties of the task environment, such as cues or predictability, may influence preparation processes, current evidence does not provide strong support for predictability as a robust moderator of age differences in switching efficiency.

While there is evidence that older adults are slower at task-switching than young adults (Wasylyshyn et al., 2011), a recent meta-analysis (Chen & Hsieh, 2023) suggested that the evidence is insufficient to confirm that local-switch costs are significantly larger in older adults. This may be because age-related decline in task-switching may be expressed differently with different switch-rule categories. For example, in voluntary switching, age-related decline could be compensated for by voluntarily choosing to minimise the amount of switching, and thus prioritising task-performance. When memorising switch-rules for the whole trial block, age-related decline in task-switching may be confounded by the influence of age-related memory decline (Greene & Naveh-Benjamin, 2023), as noted by Kray et al. (2010) and Schapkin et al. (2014). One way to minimise the confounds of voluntary choice and memory is to cue before each trial of the task. Pre-task cues serve to prompt participants to prepare for task-switching, and thus may improve task-switching performance for both young and older adults. However, compared to young adults, older adults who demonstrate a tendency to prioritise accuracy over speed may be more likely to benefit from this opportunity to prepare in advance for task-switching. Overall, before being able to examine age-related decline, variations in how age-related decline in task-switching performance can be manifested necessitates an investigation into the effects of different switch-rules on task-switching performance in older adults.

### Sample size and effect size in task-switching paradigms

The relationship between sample size and effect size can be considered crucial in shaping the robustness and generalizability of findings in the task-switching paradigm literature. Cognitive psychology studies often have small sample sizes, which can lead to biases from unreliable or inconsistent statistical outcomes (Cao et al., 2024). Therefore, in synthesising the evidence from the task-switching literature, it is important to assess whether statistically significant or non-significant effects are the result of a small or large sample size.

There are a number of narrative reviews and meta-analyses regarding task-switching (e.g., Koch et al., 2018). Vandierendonck and colleagues (2010) investigated the underlying mechanisms of task-switching in their review. Around the same time, Wasylyshyn et al. (2011) conducted a meta-analysis focussing on task-switching in ageing. Recently, Chen and Hsieh (2023) published an updated and extended meta-analysis on the same topic. Although previous reviews have examined the sources of such variability, including differences in paradigm characteristics (e.g., predictable *versus* unpredictable switching; Chen & Hsieh, 2023), the current review aims to provide a more systematic synthesis of paradigm-related factors that may contribute to variability in task-switching performance. Therefore, the current systematic review aims to bolster our understanding of how task-switching performance in healthy older adults is influenced by paradigm manipulations: the choice of base-task and switch-rule; the complexity of stimulus-features; the informativeness of task-environment; and preparation-time, including time interval between trials and time interval between cue and stimulus. An understanding of how the task-switching paradigm has changed over time, and a consideration of the impact of these changes in studies comparing performance in young and older adults, is required to provide context to the exploration of age-associated changes in task-switching performance.

## Methods

### Search protocol

This systematic review was conducted following the Preferred Reporting of Items for Systematic Reviews and Meta-Analyses (PRISMA) guidelines (Page et al., 2021). The search was performed in the three largest databases for psychology and medical science (Paperpile, 2021): Ovid, Web of Science (WoS), and Scopus. The original search was undertaken on 28/08/2021 and updated on 22/08/2023 and 21/03/2026. Both updates were performed in Ovid, WoS, Scopus and PubMed. Search terms are presented in Table 1. Title screening was undertaken by XH. Abstracts were screened by XH and EIW, who each reviewed a different set of abstracts. All full texts were screened by XH and EIW independently, with discrepancies resolved by AAD. Data was extracted by EIW and XH, with independent verification by AAD.

**Table 1:** List of search terms

Categories	Search Terms
Task-Switching Keywords	(Task-Switching OR Task Switching OR Set-Shifting OR Set Shifting OR Task-shifting OR Task shifting OR Set-Switching OR Set Switching OR Cognitive-Switching OR Cognitive Switching OR Cognitive-Shifting OR Cognitive Shifting OR Cognitive-Flexibility OR Cognitive Flexibility)
Ageing Keywords	AND (Late* Life OR Aging OR Ageing OR Old* Adult* OR Old old OR Old-old)
Primary Exclusion Keywords	AND NOT (Medica* OR Intervent* OR Brain Damage OR Cognitive Impairment OR Child* OR Adoles* OR Teen* OR Young* adult* OR Middle Age)
Secondary Exclusion Keywords	AND NOT (Damage OR Impairment OR Disease OR Hospit* OR Disab* OR Drug OR Substance OR Physiolog* OR Syndrome OR Animal OR Mouse OR Mice OR Symptom OR Stroke OR Neglect OR Trum* OR Patient OR Brain Injury OR Schizophrenia OR Lesion OR Bipolar)

The secondary exclusion keywords were searched in the title, abstract and keyword field to avoid over exclusion. All other search terms were searched in all fields to include all of the relevant results. The plural forms of single word search terms were automatically included by search mechanisms of the databases; hence truncation was not used on those plural terms.

### Inclusion and exclusion criteria

At the title-screening stage, articles were excluded if they:

1. were written in a language other than English;
2. did not include at least one group or sub-group of participants aged 60 or older (following the cut-off definition of “older adults” used by World Health Organisation WHO, 2021)^1^;
3. were review or commentary papers rather than first-hand data collection papers;
4. were unrelated to the general topic of task-switching;
5. did not include human participants;
6. included participants with physical conditions known to damage the brain (e.g., stroke, epilepsy);
7. focussed primarily on groups of participants with psychological conditions associated with acute executive dysfunction and damage to the brain (e.g., schizophrenia, substance abuse)^2^;
8. included experimental manipulation of natural cognitive performance, for example using medication or a cognitive training intervention.

At the abstract screening stage, the same criteria as for title screening were applied but additionally, articles were excluded if they:

(9) did not include task-switching related experimental paradigms or measures;
(10) were not available, either as an abstract or full-text.

At the full-text screening stage, the same criteria as for abstract screening were applied, with a final inclusion judgement based on whether the article:

(11) explicitly included the task-switching paradigm, or a variant of the task-switching paradigm. Journal articles that used other tests or paradigms that could be regarded as measures of cognitive switching ability (e.g., Wisconsin Card Sorting Test, Trail-Making Test, Stroop Task, Go-No-Go Task) were excluded.

### Data extraction and study quality

Data was extracted in six main sections, explained in full in the Supplementary Material (Table 1). Briefly, these were: Basic Journal Article Information (e.g., year of publication); Experimental Paradigm Information (e.g., type of task-switching task); Additional Relevant Information (e.g., participant characteristics including age of older participants, and where applicable, young participants included as a comparison group); Longitudinal Design Information (e.g., number of repeated measures); Task-Switching Definition Information (e.g., proposed theoretical source of switch costs); and Switch Cost Information (such as effect size of switch cost in young and older adults). For studies not reporting effect sizes, but including relevant information (e.g., *F*-statistics and degrees of freedom for ANOVA), an estimation of partial eta-squared was calculated using R (Version 4.3.2), RStudio (Version 2023.09.1), and function supplied by EIW (See Supplementary Material R Code Section). Sample size evaluation technique developed by Garner et al. (2022) was used to assess potential bias in effect size relative to sample size. Study quality was assessed by the Meta Quality Appraisal Tool (MetaQAT, Rosella et al., 2016), chosen due to its applicability to multiple types of studies, including randomised control trials, cohort studies, cross sectional studies, mixed methods research, and qualitative research.

In the Results and Discussion sections of the systematic review, we adopted a numbering system (e.g., [Study 66]; see Supplementary Table 2) to reference the studies reviewed. This was intended to enhance the clarity and transparency of reporting.

## Results

**Fig. 2:**
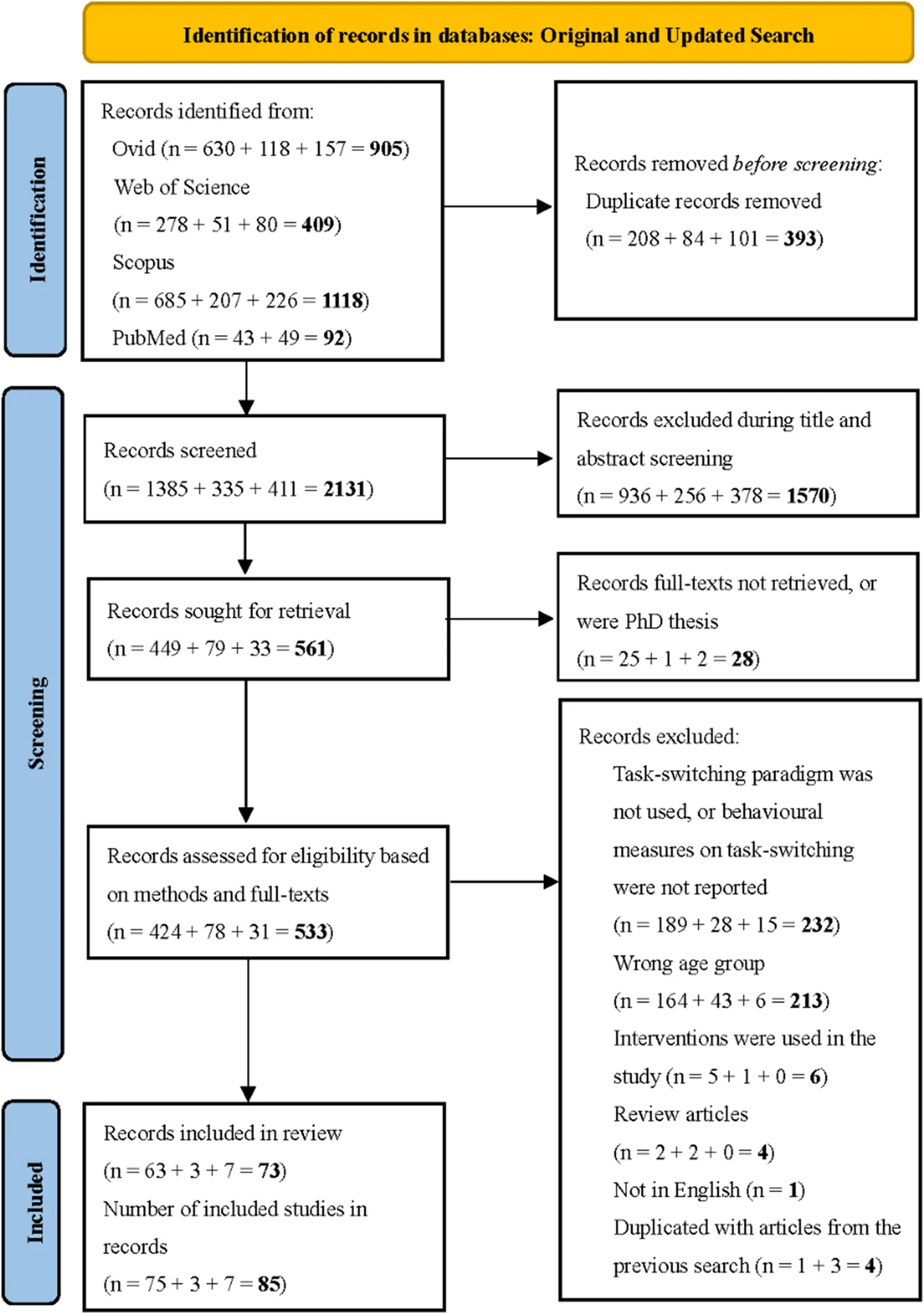
PRISMA diagram for the process of identification, screening and inclusion. Note. n represents the total number of records screened, calculated by adding together the number of records identified in the original search and the number of additional records identified through subsequent search updates.

As summarised in Fig. 2, the original search returned a total of 1593 records, the first updated search returned a total of 419 records, and the second updated search returned a total of 512 records. After title, abstract, and full-text screening, a total of 73 publications were included in the current systematic review. As some publications included multiple studies, there were 85 studies included. No study was excluded due to an unacceptable level of poor quality according to our evaluations based on guidelines provided by MetaQAT (Rosella et al., 2016).

### Basic study information

Selected publications were published between 1997 and 2026, with a peak number of 9 publications published in 2015. The number of publications on task-switching in healthy older adults increased within the period of 2012 to 2016 (see Supplementary Material Fig. 1). The average total participant sample size was *N* = 67.07 (range of *N* = 16 to *N* = 369). To investigate the age-related differences in task-switching function, a large proportion of the studies reviewed (71 out of 85) applied the design of comparing older adult groups with young adult groups. These sub-groups ranged from *N* = 9 to *N* = 124 (young) and *N* = 8 to *N* = 245 (older). The methods of reporting age characteristics varied. Of the 85 studies, 75 reported the mean age for older adults, which ranged from 62.9 to 80.3, and 59 of these studies also reported standard deviations (*SD*), which ranged from 0.34 to 9.38. Sixty-one out of the 85 studies also reported the age range for older adults, and the minimum age for older adults was 59, and the maximum age was 97. Studies that used young adults as a comparison group reported average ages ranging from 18.4 to 32.1 (*SD* range = 0.90 to 8.20, minimum age = 17, maximum age = 40).

Of the 85 studies, 67 reported the participant sample’s gender characteristics. Studies disproportionately included women. One study (Gajewski & Falkenstein, 2015) included only men. Of the 66 studies including both genders, the average proportion of women was 57.85%, ranging from 35.94% to 82.05% (mean 57.79%, range 36.36% to 87.50% for older adult group; mean 57.17%, range: 29.41% to 95.00% for young adult comparison group).

### Measurement of switch costs

#### Switch costs across age groups

Switch costs were not operationalised in the same way across studies. As shown in Table 2, the most common measures were local-switch costs (i.e., RT and accuracy differences between no-switch trials and switch trials) and global-switch costs (i.e., RT and accuracy differences between pure-block trials and no-switch trials in the mixed-blocks). No-switch trials were performed faster and with higher accuracy compared to switch trials, and pure-block trials were performed faster and with higher accuracy compared to no-switch trials in mixed-blocks.

**Table 2:** Measurement of switch costs

Switch Cost Names	Definitions	Measurement Types	Publication Count	Reports of Testing Count	Significance Count (%)
Local-Switch Costs (Switching Cost, Specific Switch Cost)	The difference between the performance of switch trials and no-switch trials in mixed-blocks.	Reaction Time	31	38	**34** (89.47%)
		Accuracy/Error Rate	19	21	**21** (100%)
		Log Reaction Time	3	3	**3** (100%)
		Drift Rate^a^	1	1	**1** (100%)
		Non-Decision Time^a^	1	1	**1** (100%)
Global-Switch Costs (Mixing Cost, General Switch Cost)	The difference between the performance of mixed-block and single task block.	Reaction Time	24	24	**24** (100%)
		Accuracy/Error Rate	18	19	**16** (84.21%)
		Log Reaction Time	1	1	**1** (100%)
Alternate-Switch Costs	The difference between the performance of alternation block and single task block.	Reaction Time	3	4	**4** (100%)
		Accuracy/Error Rate	3	3	**3** (100%)
N-2-Repetition Costs	The difference between the performance of N-2 repetition trials and N-2 switch trials.	Reaction Time	1	1	**0** (0%)
		Accuracy/Error Rate	1	1	**0** (0%)
Predictable-Shift Costs	The difference between the performance of cue-predicted switch trials and non-switch trials.	Reaction Time	1	1	**1** (100%)
		Accuracy/Error Rate	1	1	**0** (0%)
Unpredictable-Shift Costs	The difference between the performance of cue-unpredicted switch trials and non-switch trials.	Reaction Time	1	1	**1** (100%)
		Accuracy/Error Rate	1	1	**0** (0%)

Publication Count: Number of manuscripts using this type of switch-cost measure. Reports of Testing Count: Number of reported statistical significance test results for this type of switch-cost. These numbers did not add up to the total of 85 studies, as some studies implemented more than one switch-cost measure. Significance Count (%): Out of the Reports of Testing Count, number of statistically significant switch costs in this type of switch cost measure, and its percentage out of the total numbers of reports. ^a^: Outcome of the diffusion model analysis, see Ging-Jehli and Ratcliff (2020) for more details.

Other switch costs reported included N-2-repetition costs and alternate-switch costs. In Lawo et al. (2012 [Study 1278]), N-2-switched trials (i.e., Task-Cs in task sequence ABC) were performed faster and with higher accuracy compared to N-2-repeated trials (i.e., Task-Cs in task sequence CBC). Some studies (e.g., Hirsch et al., 2016 [Study 128]) used the alternate switch-rule, in which participants switch from the previous-task (e.g., Task A) to the alternative-task (e.g., Task B) between each trial for the whole trial block. Here, the outcome was the alternate-switch cost, which was the reaction time and/or accuracy differences between performance on the pure-block trials and performance on the alternate-block trials where they switch between Task A and Task B. Pure-block trials were performed faster and with higher accuracy compared to alternate-block trials.

There was also one study that used a predictable-shift cost and an unpredictable-shift cost (Van Asselen & Ridderinkhof, 2000 [Study 1036]). The predictable-shift cost was the RT and accuracy differences between the cue-predicted-switch trials (i.e., the pre-task cue provided correct information for the following trial) and no-switch trials. The unpredictable-shift cost was the RT and accuracy differences between the cue-unpredicted-switch trials (i.e., the pre-task cue provided wrong information for the following trial) and no-switch trials. No-switch trials were performed faster compared to both cue-predicted-switch trials and cue-unpredicted switch trials. Furthermore, cue-predicted-switch trials were performed faster compared to cue-unpredicted switch trials.

Nagy et al. (2023) [Study 1550] used a unique paradigm manipulation. In their design, both the task (colour-comparison or shape-comparison) and the focal stimuli (inner or outer of a shape) can be switched. In both young and older adults, RT was significantly shorter in no-switch trials compared to inner-outer-switch trials (i.e., the focal stimuli switched from the inner shape to the outer shape, or vice versa), and inner-outer-switch trials had shorter RTs than double-switch trials (i.e., both the focal stimuli and the task – colour-comparison or shape-comparison – were switched). Additionally, double-switch trials had significantly lower error rates and significantly larger switch costs, compared to the inner-outer-switch trials.

#### Switch costs in older adults

Although all studies included in this systematic review involved participants aged 60 years and older, 14 studies recruited older adults exclusively and examined task-switching performance only within this age group. One out of the 14 studies (Verstynen et al., 2012) [Study 271] investigated age as a continuous variable within their sample of older adults, and found that older age was associated with reduced performance accuracy. This reduced accuracy was found both in general task performance and in switch performance. Seventy-one out of the reported 85 studies investigated age-related differences in task-switching by comparing young adult groups with older adult groups. Among the selected studies, information related to age-group comparisons was mainly reported by two different approaches. The first approach reported the significance of interactions between age group and switch conditions. The second approach reported the global and local switch characteristics of each specific age group. Other approaches were excluded in the result synthesis, these included making direct comparisons of raw RT and accuracy between young and older adults, and analysis of performance on pure block trials, no-switch trials and switch trials together. These approaches were excluded because they do not directly isolate the cost of switching, and therefore provide less direct evidence for evaluating age-related differences in switch costs. For switch costs measured by RT: 20 out of 27 studies reported significantly larger local-switch costs in older adults compared to young adults; 18 out of 19 studies reported significantly larger global-switch costs in older adults compared to young adults. For switch costs measured by accuracy: 8 out of 12 studies found significantly larger-local-switch costs in older adults compared to young adults; 6 out of 9 studies found significantly larger global-switch costs in older adults compared to young adults. Van Asselen and Ridderinkhof (2000) reported a speed-accuracy trade-off in older adults, with faster responses accompanied by lower accuracy. However, this relationship was not explicitly examined in most of the studies reviewed. Thus, the available evidence does not allow us to determine whether speed-accuracy trade-offs are a consistent feature of task-switching performance in older adults.

To investigate potential age-related differences further, we assessed studies reporting local-switch costs measured by RT for the most frequently used base-task and switch-rule categories, namely digit base-tasks and word-cued switch-rules. However, the available evidence was limited and heterogeneous. Among the 15 studies using digit base-tasks, only 7 reported the relevant local-switch RT outcome, and these studies differed substantially in age-group composition, with some including both young and older adults while others included older adults only. Similarly, among the 26 studies using word-cued switch-rules, only 11 reported the local-switch costs measured by RT. These studies varied in both the size and composition of the age groups, and in the level of detail with which participants’ ages were reported. Some studies showed overlap in reported age ranges, and the potential overlap between samples could not be reliably established from the published information. In addition, several studies did not provide sufficient statistical information to support effect-size estimation or quantitative comparisons. Given that the overall available evidence was insufficiently comparable to support a meaningful quantitative synthesis of age-related differences, we have instead reported the patterns of age-related differences descriptively.

Taken together, the pattern of age-related differences across the 85 selected studies was inconsistent. This lack of a consistent pattern may reflect the considerable variation in experimental manipulations, including the base-task category used, the switch-rule employed, and whether switch cost was operationalised as local-switch or global-switch costs, and measured by RT or accuracy. Given this degree of methodological heterogeneity, findings across studies are difficult to relate to one another directly, and the absence of a consistent age-related pattern in switch costs may reflect this variability in paradigm design as much as it reflects variability in the underlying age-related effect.

### Paradigm characteristics

#### Base-tasks

**Fig. 3:**
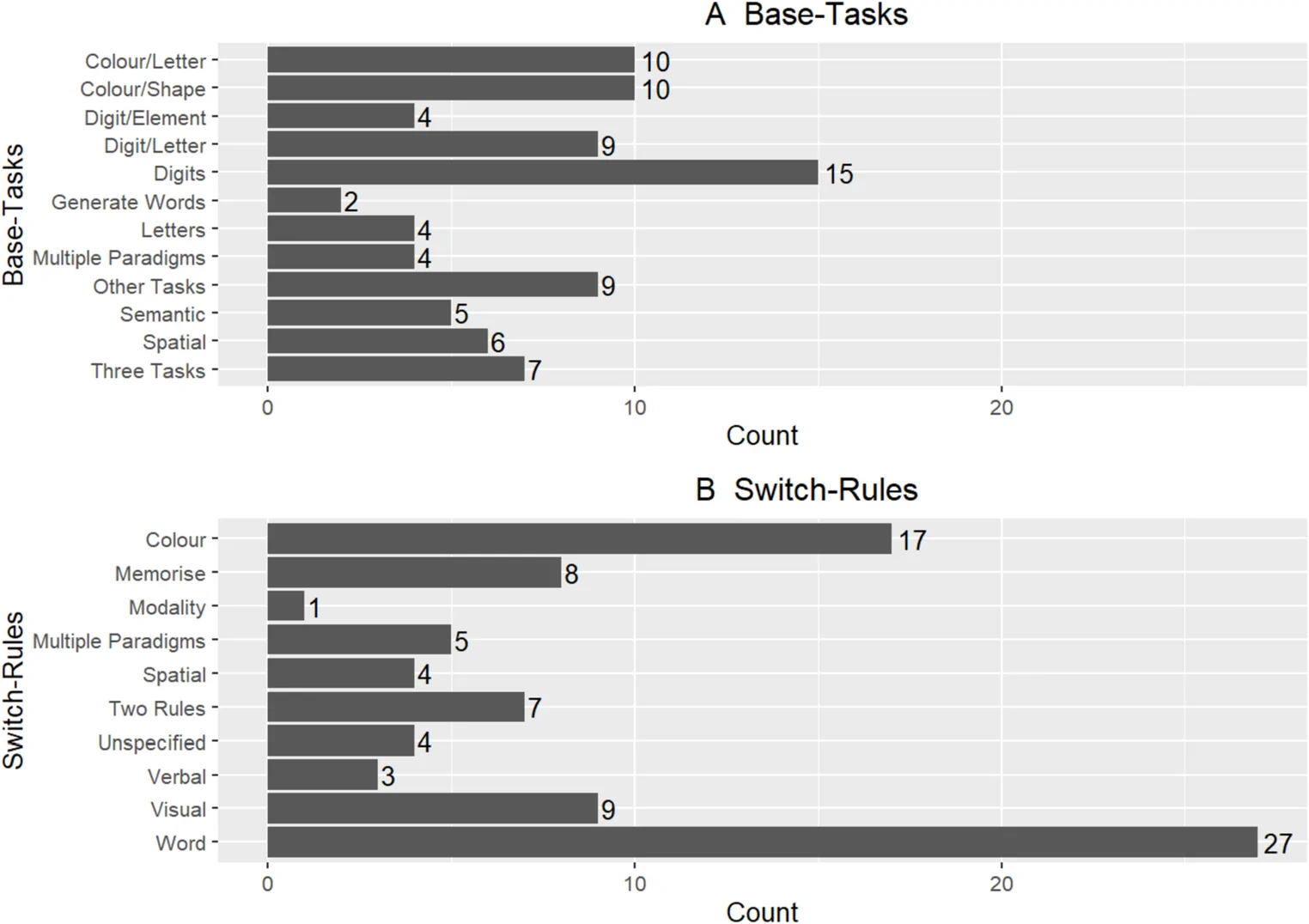
Graph of base-tasks and switch-rules for 85 studies. Note. Count = The number of studies reviewed in each of the base-task/switch-rule categories. Three Tasks = Switch between three tasks, shortened for better visualisation. Unique Task = Task unique to a single study, shortened for better visualisation.

We organised the base-tasks in identified studies into twelve categories: colour and letter, colour and shape, digit and element number, digit and letter, digits, generate words, letters, multiple task-switching paradigms, other base-task unique to a single study, semantic and non-semantic, spatial, and switch between three base-tasks. As illustrated in Fig. 3, the most common base-task was a switch between digits—digit-parity and digit-comparison tasks. Of the 85 studies, 77 studies required participants to switch between two base-tasks, and the remaining 8 out of 85 studies required participants to switch between three base-tasks. There has been an increase in variation and complexity in the base-tasks of the task-switching paradigm over time; all four of the studies that used multiple task-switching paradigms were published after 2010, and all nine of the studies with unique base-tasks were published after 2007.

##### Comparisons of Switch Cost Related Findings Across Base-Task Categories

Switch costs across different base-tasks are summarised in Table 3. Analysis and reporting varied according to base-task categories. Seven of the base-task categories (digits, colour and shape, colour and letter, switch between three base-tasks, spatial, semantic and non-semantic, and multiple task-switching paradigms) focused more on reporting local-switch costs, specifically on the significant findings^3^ for local-switch costs. No-switch trials were performed significantly faster and/or with significantly higher accuracy compared to switch trials. Some non-significant local-switch costs were reported in the colour and shape base-tasks (Kunimi et al., 2016 [Study 732]; Zhu et al., 2015 [Study 963]), and the semantic and non-semantic base-tasks (Madden et al., 2008 [Study 272]). Finally, statistical significance test results specific to switch costs were not reported for the word-generation base-tasks. The remaining four base-task categories (digit and letter, spatial, digit and element number, letter, generate words, and base-task unique to a single study) focused more on reporting global-switch costs (1 digit and letter base-task, 1 digit and element number base-task, 1 letter base-task, and 1 base-task unique to a single study). Similar to local-switch costs, in studies reporting global-switch costs, most of the studies found statistically significant global-switch costs; however, non-significant global-switch costs were found in the study switching between three base-tasks (Rey-Mermet & Meier, 2015 [Study 66]).

**Table 3:** Comparison of switch cost related findings across base-task categories

Base-Task Category (*N* = Number of studies)	Local (RT)	Local (Accuracy)	Global (RT)	Global (Accuracy)	Other Related Findings
Digit tasks (*N* = 15)	Sig = 8 Non-sig = 0	Sig = 4 Non-sig = 0	Sig = 5 Non-sig = 0	Sig = 2 Non-sig = 0	Significant alternate-switch costs measured by RT were identified (Hirsch et al., 2016 [Study 128]).
Colour and Shape tasks (*N* = 10)	Sig = 3 Non-sig = 2	Sig = 2 Non-sig = 2	Sig = 1 Non-sig = 0	Sig = 0 Non-sig = 0	Significant alternate-switch costs measured by RT and accuracy were identified (Hakun et al., 2015 [Study 540]).
Colour and Letter tasks (*N* = 10)	Sig = 6 Non-sig = 2	Sig = 3 Non-sig = 0	Sig = 3 Non-sig = 0	Sig = 1 Non-sig = 0	—
Digit and Letter tasks (*N* = 9)	Sig = 2 Non-sig = 0	Sig = 1 Non-sig = 0	Sig = 4 Non-sig = 0	Sig = 3 Non-sig = 0	Significant alternate-switch costs measured by RT and accuracy were identified (Hirsch et al., 2016 [Study 128]). Significant predictable-shift costs and unpredictable-shift costs measured by RT were identified (Van Asselen & Ridderinkhof, 2000 [Study 1036]).
Switch between three tasks (*N* = 7)	Sig = 3 Non-sig = 0	Sig = 2 Non-sig = 0	Sig = 2 Non-sig = 0	Sig = 2 Non-sig = 2	Age-specific global-switch costs were identified in both young and older group (Rey-Mermet & Meier, 2015 [Study 66]). N-2-repetition costs measured by RT and accuracy were not significant (Lawo et al., 2012 [Study 1278]).
Spatial tasks (*N* = 6)	Sig = 3 Non-sig = 0	Sig = 4 Non-sig = 0	Sig = 1 Non-sig = 0	Sig = 2 Non-sig = 0	Significant global- and local-switch costs measured by RT latencies were identified (Bojko et al., 2004 [Study 88]).
Semantic and Non-Semantic tasks (*N* = 5)	Sig = 0 Non-sig = 1	Sig = 1 Non-sig = 0	Sig = 0 Non-sig = 0	Sig = 0 Non-sig = 0	Significant global- and local-switch costs measured by log RT latencies were identified (Kray & Eppinger, 2006 [Study 494]).
Digit and Element number tasks (*N* = 4)	Sig = 1 Non-sig = 0	Sig = 0 Non-sig = 0	Sig = 1 Non-sig = 0	Sig = 1 Non-sig = 0	—
Letter tasks (*N* = 4)	Sig = 0 Non-sig = 0	Sig = 0 Non-sig = 0	Sig = 2 Non-sig = 0	Sig = 2 Non-sig = 0	—
Word-Generation tasks (*N* = 2)	Sig = 0 Non-sig = 0	Sig = 0 Non-sig = 0	Sig = 0 Non-sig = 0	Sig = 0 Non-sig = 0	No test specific to switch costs was recorded. Studies suggested older adults have a higher rate of failure with switching word categories than young adults (Mayr & Kliegl, 2000 [Study 341]).
Task unique to a single study (*N* = 9)	Sig = 4 Non-sig = 0	Sig = 2 Non-sig = 0	Sig = 3 Non-sig = 0	Sig = 2 Non-sig = 1	Significant alternate-switch costs measured by RT and accuracy were identified (Verhaeghen & Hoyer, 2007 [Study 162]). Pettigrew and Martin (2016) [Study 1281] identified larger N-2-repetition costs in older adults, but this was not specific to switch costs. Double-switch trials had significantly larger switch costs, compared to the inner-outer-switch trials (Nagy et al., 2023 [Study 1550]).
Multiple Paradigms (*N* = 4)	Sig = 3 Non-sig = 0	Sig = 0 Non-sig = 0	Sig = 0 Non-sig = 0	Sig = 0 Non-sig = 0	In Albinet et al. (2012) [Study 943], results were reported in the form of composite scores. This study found that older age was related to worse shifting composite scores (task-switching paradigm was one of the paradigms used to generate this score).

More details on base-tasks can be found in the Supplementary Material Table 3 (Base-tasks summary). Details on the definitions of switch cost can be found in Table 2 (Switch-cost summary). Dash represents that there is no information available. RT = Reaction Time. Local = local-switch costs. Global = global-switch costs. Sig = statistically significant findings. Non-sig = statistically non-significant findings. The availability of studies differed across base-task categories, resulting in unequal representation of the categories in the literature.

Other switch costs (defined in Table 2) were used with different base-tasks. Significant alternate-switch costs measured by RT was found in a study using the digit base-task (Hirsch et al., 2016 [Study 128-1]), and measured by both RT and accuracy in studies using the colour and shape base-task (Hakun et al., 2015 [Study 540]), the digit and letter base-task (Hirsch et al., 2016 [Study 128-2]), and the base-task unique to a single study (Verhaeghen & Hoyer, 2007 [Study 162]). Both a significant predictable-shift cost and an unpredictable-shift cost were found by Van Asselen and Ridderinkhof (2000) [Study 1036] for the digit and letter base-task.

##### Effect Sizes

Effect-size information was not consistently available across the studies reviewed. For the purpose of conducting adequate summarisation, effect sizes were extracted or calculated only for the studies that used ANOVA and provided sufficient statistical information for their calculation. The available effect-size data therefore represents only a subset of the studies, statistical tests, and outcomes included in this systematic review. These data comprised both effect sizes reported directly by the original studies and effect sizes calculated from the statistical information provided in the published reports. Of all the studies reviewed, the following information was calculated based on available information: 54% local-switch costs measured by RT, 50% local-switch costs measured by accuracy, 47% global-switch costs measured by RT, and 58% global-switch costs measured by accuracy. Most of the studies that reported non-significant findings did not provide sufficient statistical information to calculate effect sizes, resulting in relatively few effect size estimations for non-significant effects. As the studies included in this systematic review generally had modest sample sizes, and the effect-size estimates were more likely to be observed or reported when statistical significance was reached, the available effect-size data may over-represent larger effects. Consequently, summaries based on the available effect sizes may overestimate the magnitude of switch costs across the studies reviewed. For this reason, we considered effect sizes and statistical significance as separate but complementary components of the synthesis rather than integrating them into a single comparison.

**Fig. 4:**
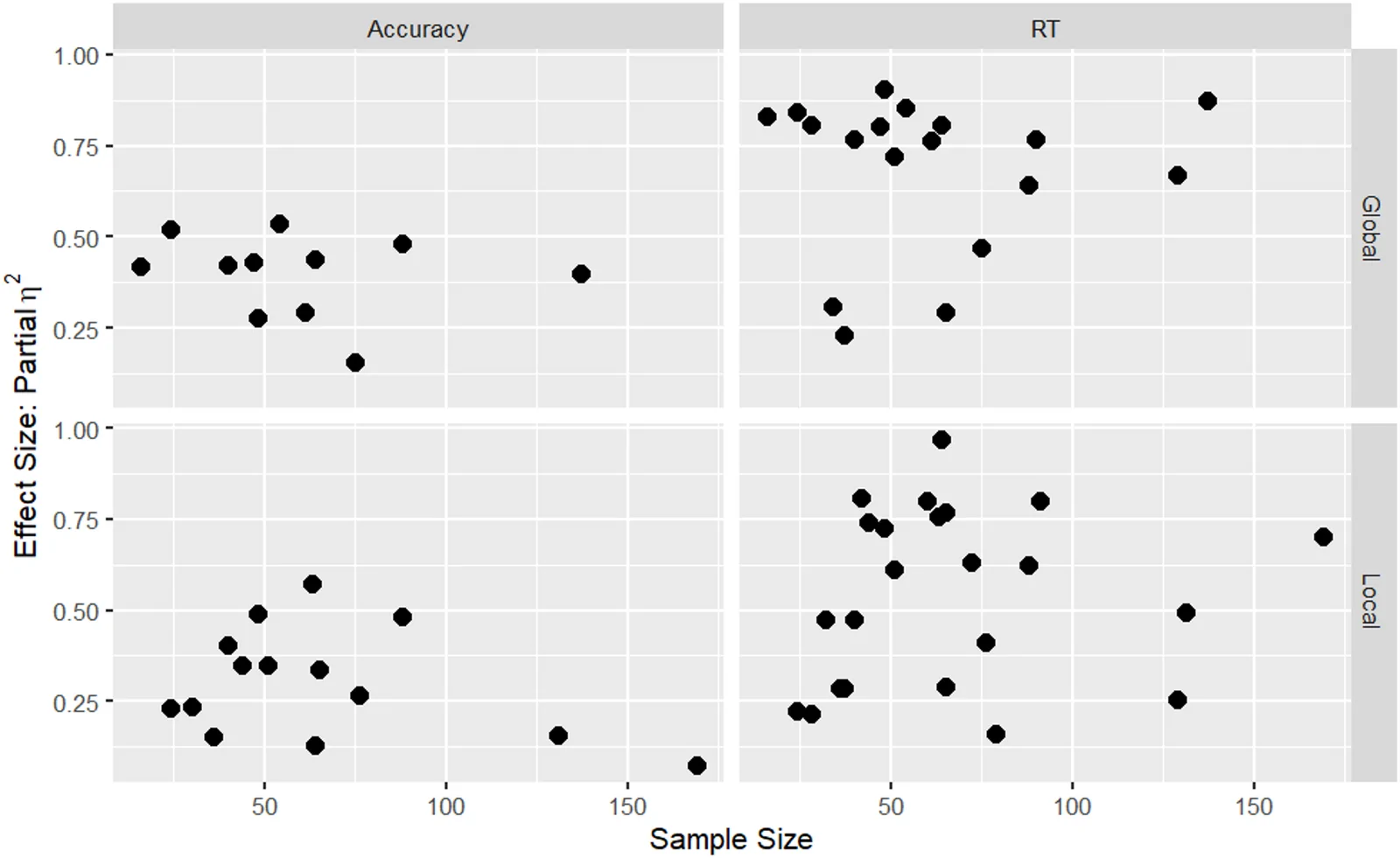
Relationship between effect sizes and sample sizes. Note. This figure depicts the relationship between effect sizes and study sample sizes. The pattern indicates a lack of bias in effect size related to sample size in the studies reviewed, per Garner et al. (2022). Accuracy = switch costs measured by accuracy. RT = switch costs measured by reaction time. Global = global-switch costs. Local = local-switch costs.

Switch-cost effect sizes, as represented by partial eta-squared (η^2^) , are summarised in the Supplementary Materials (Tables 4, 5, 6 and 7; Figs. 2, 3, 4 and 5). Of the 80 incidences where effect sizes were reported or could be estimated via partial η^2^, 46 analysed local-switch costs, and 34 analysed global-switch costs. Mean effect sizes were larger for RT (local-switch costs: 0.54, global-switch costs: 0.71) than accuracy (local-switch costs: 0.31, global-switch costs: 0.38).

Effect sizes of local-switch costs measured by RT varied largely across studies, with a mean partial η^2^ of 0.53 (range = 0.11 to 0.97). The digit base-tasks and spatial base-tasks had relatively larger effect sizes compared to other base-tasks (mean partial η^2^ = 0.73 and 0.70 respectively). Conversely, colour and letter base-tasks had relatively smaller effect sizes (mean partial η^2^ = 0.27).

Compared to effect sizes of local-switch costs measured by RT, effect sizes of local-switch costs measured by response accuracy were smaller, and less variable, with an overall mean partial η^2^ of 0.31 (range = 0.07 to 0.57). Like effect sizes of local-switch costs measured by RT, the digit base-tasks had relatively larger effect sizes compared to other base-tasks (mean partial η^2^ = 0.40), but one study using this base-task also had the smallest reported local-switch costs effect size measured by accuracy, partial η^2^ = 0.13 (Tsai & Wang, 2015 [Study 570]). Colour and letter base-tasks had relatively smaller effect sizes (mean partial η^2^ = 0.22).

Effect sizes of global-switch costs measured by RT varied largely across studies, with a mean partial η^2^ of 0.71 (range = 0.29 to 0.93). The digit base-tasks and letter base-tasks had relatively larger effect sizes compared to other base-tasks (mean partial η^2^ = 0.81 and 0.86 respectively). Conversely, studies using paradigm switching between digit and letter base-tasks had relatively smaller effect sizes (mean partial η^2^ = 0.45). However, when compared to local-switch costs measured by RT, global-switch costs measured by RT had relatively larger effect sizes, and were less variable.

Compared to effect sizes of local-switch costs measured by accuracy, effect sizes of global-switch costs measured by accuracy had a larger range (i.e., more variation). Compared to effect sizes of global-switch costs measured by RT, effect sizes of global-switch costs measured by accuracy were smaller. Effect sizes of global-switch costs measured by accuracy also varied largely across studies, with a mean partial η^2^ of 0.38 (range = 0.16 to 0.54). The letter base-task had relatively larger effect sizes compared to other base-tasks (mean partial η^2^ = 0.47). However, of the 9 base-task categories, 5 categories (colour and letter, colour and shape, digit and element number, letter and position, spatial) included only one study, and the other 4 categories (digit, digit and letter, letters, three base-tasks) included only two studies. Hence, a summary of the relationship between base-tasks and effect sizes of global-switch costs measured by accuracy could not be constructed.

##### Base-Tasks Summary

Overall, the findings indicated large variations in the base-tasks used in the task-switching paradigms; albeit most studies involved switching between two base-tasks and they most frequently used digit-based tasks. Across the studies using different base-task categories, switch costs were generally reported as statistically significant, but with local-switch costs more commonly examined than global-switch costs. Where effect sizes were available, their magnitude varied considerably across studies and tended to be larger for RT measures than for accuracy measures. However, direct comparisons of effect magnitudes across the base-task categories are limited by the relatively small number of studies in several of the base-task categories, and also by the inconsistent reporting of effect sizes.

#### Switch-rules

Switch-rules describe the ways in which participants are instructed about the task switch required in a particular trial. We identified nine categories of switch-rules in the selected 73 records (85 studies) and a category in which the switch-rules were unspecified (4 studies), as presented in Fig. 3. Switch-rules were mainly manipulated on two aspects: switch type (switching voluntarily, memorising the switch-rules for the whole trial block, or cueing on a trial-by-trial basis), and format of the cue (e.g., word, colour, visually, spatially, verbally).

Voluntary switch-rules (2 studies) are intended to mimic naturalistic switching performance, as in Butler and Weywadt (2013) and Terry and Sliwinski (2012) [Study 85 and 149]. Memorise switch-rules (8 studies) ask participants to alternate tasks after n^th^ trial, and explore the impact of memory ability on task-switching performance. Four out of the eight studies asked participants to alternate between two tasks (Hawkes et al., 2012; Hirsch et al., 2016; Verhaeghen & Hoyer, 2007 [Study 128, 1370, and 162]), two studies reported by Rey-Mermet and Meier (2015) [Study 66] presented tasks in triplets, and the remaining two studies asked participants to switch tasks every third trial (Gajewski & Falkenstein, 2015; Simon & Bock, 2017 [Study 763 and 1267]).

Cueing aims to remove the impact of memory on task-switching performance, by either cueing before trial onset (de Bruin et al. 2015a [Study 545]) or simultaneously with the trial stimulus (Ging-Jehli & Ratcliff, 2020 [Study 489]). Cues can take several forms (word, colour, visually, spatially, verbally). Word cues (27 studies), where words or abbreviations of words were presented as cues for performing the corresponding task (e.g., “colour” for the colour-discrimination task), were the most common. Colour cues (17 studies), where the desired switch-rule was associated with a colour, were the second most common. Visual cues (9 studies), where the desired switch-rule was associated with a shape or gradient. Spatial cues (4 studies), where the location of the stimulus on the screen (typically upper or lower half) indicated the switch task (Erickson, 2008; Glisky et al., 2021; Kunimi et al., 2016; Mulligan et al., 2020 [Study 30, 401, 627, 732]). Verbal cues, where verbal instructions were presented as cues for performing the corresponding tasks, was found in three studies (two studies reported in Mayr & Kliegl, 2000 [Study 341], Mayr, 2001 [Study 82]). In one study, a simultaneous luminance and word cue was used (Ging-Jehli & Ratcliff, 2020 [Study 489]).

Some of the reviewed studies tested and compared multiple switch-rules. Seven studies contrasted two switch-rules. Comparisons of colour-cued *versus* word-cued switching (Chang et al., 2020 [Study 121]) and visually-cued *versus* word-cued switching (Tsai & Wang, 2015 [Study 570]) found that word-cued switching was associated with lower switch costs. Voluntary, rather than word cued (Gajewski et al., 2011; Hakun et al., 2015 [Study 540, 1230]) or visually cued (Dai et al., 2013 [Study 568]) switching was associated with lower switch costs. There was further evidence that in voluntary cueing older adults chose to switch less often to maintain best task performance (Butler & Weywadt, 2013; Terry & Sliwinski, 2012 [Study 85 & 149]). Gaál and Czigler (2015) [Study 136] compared informative cues (i.e., cues that provide task-relevant information) with non-informative cues (i.e., cues that do not provide task-relevant information), and reported that participants responded quicker when the cues were informative. This was also supported by the findings of Chang et al. (2020) [Study 121].

##### Comparison of Switch Cost Related Findings Across Switch-Rule Categories

Switch costs across different switch-rules are summarised in Table 4. As with base-tasks, analysis and reporting varied. Most studies did not report statistical significance-test (i.e., ANOVA) results for global- and local-switch costs, with more studies focusing on local-switch costs, and measuring switch costs in terms of RT rather than accuracy or both RT and accuracy. In four categories of switch costs (colour-cued, double switch-rules, multiple task-switching paradigms, and verbally-cued) only the significant switch costs were reported.

**Table 4:** Comparison of switch cost related findings across switch-rule categories

Switch-Rule Category (*N* = Number of studies)	Local (RT)	Local (Accuracy)	Global (RT)	Global (Accuracy)	Other Related Findings
Words (*N* = 27)	Sig = 9 Non-sig = 3	Sig = 7 Non-sig = 0	Sig = 6 Non-sig = 0	Sig = 4 Non-sig = 0	Significant predictable-shift costs and unpredictable-shift costs measured by RT were identified (Van Asselen & Ridderinkhof, 2000 [Study 1036]). Significant global- and local-switch costs measured by RT latencies were identified (Bojko et al., 2004 [Study 88]). Significant global- and local-switch costs measured by log RT latencies were identified (Kray & Eppinger, 2006 [Study 494]). Significant N-2-repetition costs measured by RT and accuracy were identified (Lawo et al., 2012 [Study 1278]). Double-switch trials had significantly larger switch costs, compared to the inner-outer-switch trials (Nagy et al., 2023 [Study 1550]).
Colour (*N* = 17)	Sig = 6 Non-sig = 0	Sig = 3 Non-sig = 0	Sig = 8 Non-sig = 0	Sig = 7 Non-sig = 0	—
Visual (*N* = 9)	Sig = 5 Non-sig = 1	Sig = 3 Non-sig = 0	Sig = 4 Non-sig = 0	Sig = 1 Non-sig = 0	—
Memorise (*N* = 8)	Sig = 1 Non-sig = 0	Sig = 1 Non-sig = 0	Sig = 1 Non-sig = 0	Sig = 1 Non-sig = 2	Significant alternate-switch costs measured by RT and accuracy were identified (Hirsch et al., 2016 [Study 128], Verhaeghen & Hoyer, 2007 [Study 162]). Significant alternate-switch costs measured by RT were identified (Hirsch et al., 2016 [Study 128]). Significant age-specific global-switch costs were identified in both young and older groups (Rey-Mermet & Meier, 2015 [Study 66]).
Double Switch-Rules (*N* = 7)	Sig = 6 Non-sig = 0	Sig = 4 Non-sig = 0	Sig = 2 Non-sig = 0	Sig = 1 Non-sig = 0	Significant alternate-switch costs measured by RT and accuracy were identified (Hakun et al., 2015 [Study 540]).
Multiple Paradigms (*N* = 4)	Sig = 3 Non-sig = 0	Sig = 0 Non-sig = 0	Sig = 0 Non-sig = 0	Sig = 0 Non-sig = 0	In Albinet et al. (2012) [Study 943], results were reported in the form of a composite score. This study found that older age was related to a worse shifting composite score (task-switching paradigm was one of the paradigms used to generate this score).
Spatial (*N* = 4)	Sig = 0 Non-sig = 1	Sig = 0 Non-sig = 1	Sig = 0 Non-sig = 0	Sig = 0 Non-sig = 0	—
Verbal (*N* = 3)	Sig = 1 Non-sig =/	Sig = 0 Non-sig =/	Sig = 0 Non-sig = 0	Sig = 0 Non-sig = 0	—
Modality (*N* = 1)	Sig = 1 Non-sig = 0	Sig = 1 Non-sig = 0	Sig = 1 Non-sig = 0	Sig = 1 Non-sig = 0	—
Unspecified (*N* = 5)	Sig = 1 Non-sig = 0	Sig = 1 Non-sig = 0	Sig = 1 Non-sig = 0	Sig = 0 Non-sig = 1	—

Details on the definition of switch costs can be found in Table 3. Dash represents that there is no information available. RT = Reaction Time. Local = Local-switch costs. Global = Global-switch costs. Sig = statistically significant findings. Non-sig = statistically non-significant findings.

Studies of the memorise switch-rule also reported non-significant global-switch costs (Rey-Mermet & Meier, 2015 [Study 66]), which suggest that the accuracy differences between the pure-block trials and the no-switch trials in the mixed-blocks were not significant. One study from the memorise switch-rule category also reported significant alternate-switch costs measured by RT and accuracy (Verhaeghen & Hoyer, 2007 [Study 162]).

Across the 10 switch-rule categories (word-cued, visually-cued, memorise, spatially-cued, colour-cued, double switch-rules, multiple task-switching paradigms, verbally-cued, modality-cued, and unspecified switch-rules), findings varied both between and within categories. Word-cued switch-rules produced the most varied pattern of results. Several studies reported non-significant local-switch costs (Madden et al., 2008 [Study 272]; West & Moore, 2005 [Study 53]; Zhu et al., 2015 [Study 963]), whereas other studies reported significant global and local-switch costs measured by RT latencies (Bojko et al., 2004 [Study 88]) and by log RT latencies (Kray & Eppinger, 2006 [Study 494]). Significant predictable and unpredictable shift costs measured by RT were also found within this category (Van Asselen & Ridderinkhof, 2000 [Study 1036]). Additionally, Lawo et al. (2012 [Study 1278]) reported a significant N-2 repetition cost. The *N* − 2 repetition cost is typically examined by comparing performance in task sequences such as ABA and CBA. In an ABA sequence, participants return to a task that was performed two trials earlier (i.e., Task A), whereas in a CBA sequence they switch to Task A, which has not been recently performed. Performance is typically slower or less accurate in ABA sequences than in CBA sequences, resulting in the *N* − 2 repetition cost. This cost is interpreted as evidence for persisting inhibition of the previously abandoned task set. When the task must be reactivated shortly after being suppressed, residual inhibition interferes with task-set retrieval and execution. For visually-cued switch-rules, one study reported non-significant local-switch costs (West & Travers, 2008 [Study 409]), and similarily for spatially-cued switch-rules, one study reported non-significant local-switch costs (Kunimi et al., 2016 [Study 732]). For memorise switch-rules, one study reported non-significant global-switch costs (Rey-Mermet & Meier, 2015 [Study 66]). All significant findings, including those from studies using colour-cued switch-rules, double switch-rules, multiple task-switching paradigms, verbally-cued switch-rules, and modality-cued switch-rules are presented in Table 4.

##### Effect Sizes

For effect sizes of local-switch costs measured by RT, double switch-rules and visually-cued switch-rules had relatively larger effect sizes than other switch-rules (mean partial η^2^ = 0.77 and 0.63 respectively), while colour-cued and word-cued switch-rules had smaller effect sizes than other switch-rules (mean partial η^2^ = 0.41 and 0.43 respectively).

For effect sizes of local-switch costs measured by accuracy, there were no clear associations between any given switch-rule and larger effect sizes (Fig. 4), but on average visually- and word-cued switch-rules had slightly smaller effect sizes than other switch-rules (mean partial η^2^ = 0.27 and 0.29 respectively).

For effect sizes of global-switch costs measured by RT, double switch-rules had relatively larger effect sizes than other switch-rules (mean partial η^2^ = 0.87), while visually-cued switch-rules had smaller effect sizes than other switch-rules (mean partial η^2^ = 0.50), followed by colour-cued and word-cued switch-rules (mean partial η^2^ = 0.70 and 0.72 respectively), which had a pattern similar to the effect sizes of local-switch costs measured by RT and accuracy.

For effect sizes of global-switch costs measured by accuracy, visually-cued switch-rules (mean partial η^2^ = 0.52) and modality switch-rules (mean partial η^2^ = 0.48) had relatively larger effect sizes than other switch-rules. However, both of these switch-rule categories included only one study. Among switch-rule categories with multiple studies, colour-cued switch-rules had relatively larger effect sizes than other switch-rules (mean partial η^2^ = 0.40). Conversely, word-cued switch-rules had smaller effect sizes than other switch-rules (mean partial η^2^ = 0.33), which had a consistent pattern among all reported test results, regardless of whether they were local- or global-switch costs measured by RT or accuracy.

##### Switch-Rules Summary

Overall, the findings indicated there were large variations in the ways switch-rules were implemented, with cue-based switch-rules, and particularly word-cued and colour-cued rules, being the most common. Across studies using different switch-rule categories, local-switch costs were more frequently examined than global-switch costs. Most reported findings indicated significant local-switch and global-switch costs, although non-significant effects were also observed in word-cued, visually-cued, memorised, spatially-cued, modality-cued, and unspecified switch-rule categories. Double switch-rules and visually-cued switch-rules showed relatively larger effect sizes, whereas word-cued and colour-cued switch-rules generally showed relatively smaller effect sizes. However, the availability of information about effect sizes varied across switch-rule categories, with the number of studies contributing to the individual switch-rule categories varying substantially, and several categories were represented by only one or a small number of studies—thus limiting direct comparisons of effect magnitudes across switch-rule categories.

Although base-tasks and switch-rules were examined separately in the present review, these paradigm features are not entirely independent because each study combines a particular base-task with a particular switch-rule. To assess the extent to which these features were combined across the studies reviewed, we conducted an additional descriptive assessment of the base-task and switch-rule combinations represented in the most frequently used categories, namely digit base-tasks and word-cued switch-rules (See Supplementary Material: Descriptive Assessment of the Combinations of Base-Tasks and Switch-Rules). This assessment indicated substantial variation in the combinations of paradigm features used across studies. Most specific base-task and switch-rule combinations were represented by only one or a small number of studies, and the studies also varied in other methodological characteristics and outcome measures. There were insufficient numbers of sufficiently comparable studies within individual base-task and switch-rule combinations to support a meaningful quantitative examination of their interaction or to determine whether the effects associated with one paradigm feature depended on the other. We therefore retained the base-task and switch-rule analyses as separate descriptive components of the systematic review, while acknowledging that their potential effects cannot be considered entirely independent, and that the observed patterns should not be interpreted as evidence of independent effects of either feature or of their interaction.

### Effect sizes and sample sizes

For effect sizes of local-switch costs measured by RT, there was no clear linear association between effect size and study sample size (see Fig. 4), indicating these differences in effect size across base-tasks and switch-rules were not an artefact of sample size. Studies with sample size range from 42 to 64 also had relatively high effect sizes (partial η^2^ range = 0.61 to 0.96).

For effect sizes of local-switch costs measured by accuracy, there was no clear linear association between effect size and study sample size (see Fig. 4), indicating these differences in effect size across base-tasks and switch-rules were not an artefact of sample size.

For effect sizes of global-switch costs measured by RT, there was no clear linear association between effect size and study sample size (see Fig. 4), indicating that the effect of global-switch cost measured by RT was also not an artefact of large sample sizes. The peak effect size (η^2^ = 0.93) was reported in the study with a total sample size of 51. However, this peak was not directly reflected in Fig. 4, as this effect size was averaged with another study with the same total sample size of 51.

For effect sizes of global-switch costs measured by accuracy, there was no clear linear association between effect size and study sample size (see Fig. 4), indicating these differences in effect size across base-tasks and switch-rules were not an artefact of sample size.

There was no clear linear association between effect sizes and study sample sizes for either local-switch or global-switch costs measured by RT or accuracy. This suggests that the observed variation in effect sizes across studies using different base-task and switch-rule categories was unlikely to be attributable to differences in sample size. Relatively large effect sizes were also observed in studies with moderate sample sizes, further supporting the absence of a systematic sample-size effect.

### Other paradigm manipulations

Other paradigm related manipulations included stimulus-features (e.g., univalent stimulus *versus* bivalent stimulus). Bivalent stimuli can be used to perform two tasks (e.g., the stimulus “A8”, can be used to perform both a letter-discrimination task and a digit-discrimination task). Conversely, univalent stimulus can only be used to perform a single task (e.g., the stimulus “A” can be used to perform a letter-discrimination task, and the stimulus “8” can be used to perform a digit-discrimination task). Bivalent stimuli were associated with slower reaction times, and larger local-switch costs than univalent stimuli (Ging-Jehli & Ratcliff, 2020; Hirsch et al., 2016; Lien et al., 2008; Rey-Mermet & Meier, 2015 [Study 489, 127, 128, 66]). Smaller stimulus set size was associated with larger practice improvement (Kray & Eppinger, 2006 [Study 494]).

Other paradigm manipulations also included task-environment (e.g., informative cues, changed stimulus *versus* unchanged stimulus). Providing more informative cues in task-switching can result in better task-switching performance (Gaál & Czigler, 2015 [Study 136]). Cue repetition was associated with larger global-switch costs in accuracy (West & Travers, 2008 [409]). The cost of an unexpected-switch was larger than an expected-switch. The unexpected trials cost more when there was task-repetition, compared to when there was task-switching (Lien et al., 2008; Van Asselen & Ridderinkhof, 2000 [Study 127, 1036]). Overlap in response-key mapping (i.e., the keys that were used to provide responses) was associated with larger global- and local-switch costs (Mayr, 2001 [Study 82]). Additionally, when the response-key mapping as well as the task switched, similar effects of switch cost as found in task-switching was observed. There was an additive effect between response-switching and task-switching, where switching in both resulted in additional larger switch costs compared to a switch only in the response set or the task (Hahn et al., 2004 [Study 89]).

The last domain of other paradigm manipulations is preparation-time (longer *versus* shorter time intervals between the response and the next trial). Multiple measures of preparation-time were used. Longer cue-stimulus interval (CSI) was associated with smaller global-switch costs, and lower N-2-repetition costs in accuracy (Lawo et al., 2012; Meiran et al., 2001 [Study 882, 1278]). Longer inter-stimulus interval (ISI) was associated with smaller local-switch costs (Kunimi et al., 2016 [Study 732]). Longer response-cue interval (RCI) was associated with smaller local-switch costs (Meiran et al., 2001 [Study 882]). Longer response-stimulus interval (RSI) was associated with smaller local-switch costs and smaller alternate-switch costs (Hirsch et al., 2016; Kramer et al., 1999 [Study 1100, 128]).

The findings suggest that task-switching performance is sensitive to stimulus characteristics, task environment, and preparation time. Across studies, conditions that increased task complexity or included interference tended to be associated with larger switch costs, whereas conditions that provided additional preparation time or task-relevant information tended to be associated with smaller switch costs and improved performance.

### An overview of findings in relation to local-switch costs and global-switch costs

Table 5 presents the relationship between paradigm manipulations and task-switching performance measured by local-switch costs and global-switch costs. The pattern of results presented in Table 5 is summarised in the current section. However, these results need to be considered with caution, due to variation in the reporting of non-significant statistical significance-test results across studies when compared with the more consistent reporting of significant results.

**Table 5:** Overview of relationship between paradigm manipulation categories and switch costs

	Local (RT)		Local (Accuracy)		Global (RT)		Global (Accuracy)	
	Sig	Non-sig	Sig	Non-sig	Sig	Non-sig	Sig	Non-sig
**Base-Tasks**								
Colour/Letter	X	X	X		X		X	
Colour/Shape	X	X	X	X	X		X	
Digit/Element	X				X		X	
Digit/Letter	X		X		X		X	
Digits	X		X		X		X	
Letters					X		X	
Multiple Paradigms	X							
Sematic and Non-Semantic		X	X					
Spatial	X		X		X		X	
Tasks Used by One Study	X		X		X		X	X
Three Tasks	X		X		X		X	X
**Switch-Rules**								
Colour	X		X		X		X	
Double Switch-Rules	X		X		X		X	
Memorise	X		X		X		X	X
Modality	X		X		X		X	
Multiple Paradigms	X							
Spatial		X		X				
Unspecified	X		X		X			X
Verbal	X							
Visual	X	X	X		X		X	
Words	X	X	X	X	X		X	

This table presents an overview of evidence of the relationship between paradigm manipulations (i.e., base-task and switch-rule) and task-switching performance measured by local- and global-switch costs. An “X” in the corresponding column indicates there is information reported in the studies reviewed. Accuracy = switch costs measured by accuracy. RT = switch costs measured by reaction time. Global = global-switch costs. Local = local-switch costs. Sig = statistically significant findings. Non-sig = statistically non-significant findings.

In studies reporting local-switch costs, less statistical evidence (e.g., results of ANOVA) was gathered for studies that used task-switching paradigms that switched between colour and shape base-tasks, colour and letter base-tasks, semantic and non-semantic base-tasks, compared to other base-task categories. Regarding switch-rules, less statistical evidence (e.g., results of ANOVA) was gathered for studies that used word-cued, visually-cued and spatially-cued switch-rules, compared to other switch-rule categories. Within these studies, non-significant local-switch costs were reported.

In studies reporting global-switch costs, less statistical evidence (e.g., results of ANOVA) was gathered for studies that used task-switching paradigms that switched between three base-tasks, compared to other base-task categories. Regarding switch-rules, less statistical evidence (e.g., results of ANOVA) was gathered for studies that used memorise switch-rules, compared to other switch-rules categories. Within these studies, non-significant global-switch costs were reported.

## Discussion

To the best of our knowledge, this systematic review has provided the first summary of task-switching paradigm manipulations and switch costs across studies involving healthy older adults. Many of these studies also included young adult comparison groups. Although the review identified commonly used paradigm features and patterns of switch costs, the substantial heterogeneity in paradigm designs, participant samples, and reporting practices limited direct comparisons of age-related differences. The available evidence was insufficient to support a robust quantitative synthesis of differences between young and older adults for the most frequently examined paradigm categories. Therefore, the findings should be interpreted as describing patterns observed across the literature that was reviewed rather than as demonstrating that particular paradigm features produce consistent effects specifically in older adults.

In the 85 studies across 73 publications in this systematic review, we found substantial variability in how task-switching is currently measured in terms of task-switching paradigm selection, how switch costs were operationalised, and how results were reported. The switch costs were measured in six different ways (local-switch, global-switch, alternate-switch, N-2-repetition, predictable-shift, and unpredictable-shift). There were 12 base-tasks which were (from most to least common): digits; colour and shape; colour and letter; digit and letter; other base-tasks; switches between three base-tasks; spatial; semantic and non-semantic; digit and element number; letters; multiple task-switching paradigms; and generating words. There were three categories of switch-rules (from most to least common): cueing before each trial of the task; memorising switch-rules for the whole trial block; and switching voluntarily. When present, there were seven forms of cues (from most to least common): word; colour; visual; spatial; verbal; modality, and simultaneous cue-response presentation. There were also studies that used multiple switch-rules. Other within-paradigm manipulations included: stimulus-features; task-environment; and preparation time. This mirrors findings elsewhere, in that laboratory tests of cognitive constructs can vary substantially, such as experimental paradigms of short-term memory noted in Richardson (2007), inattentional blindness noted in Redlich et al. (2021), and executive function noted in Faria et al. (2015).

There was substantial variability in how results were reported, including in the precision of demographic information, and the presence *versus* absence of reporting of non-significant results. Nonetheless, findings for switch costs were somewhat consistent across studies and age groups. Local-switch costs, as measured by RT, was both the most common measure, and had the strongest support in terms of achieving statistical significance. Global-switch costs focus on the differences between the pure-block trials and the no-switch trials within mixed-blocks, which involves a switch cost alongside the higher working memory load introduced by the need to activate two tasks in a mixed-block (Kiesel et al., 2010). Working memory is another aspect of executive function that is found to decline with age (McCarrey et al., 2016), which means global-switch costs may be confounded by these age-related differences. Thus, compared to global-switch costs, local-switch costs are arguably a more appropriate focus when assessing age-related differences. When cognitive resources are not sufficient, older adults have the tendency to sacrifice RT to maintain accuracy (Van Asselen & Ridderinkhof, 2000). Due to this speed-accuracy trade-off, measures merely focussing on RT will not be sufficient to obtain a holistic picture of task-switching performance. Measures incorporating RT and accuracy (e.g., Diffusion Decision Model; Ging-Jehli & Ratcliff, 2020) are thus more appropriate for use with older adult samples.

### Switch costs—grouped by base-tasks and switch-rules

Substantial variability in the base-tasks identified in this review may have implications for whether or not statistically significant switch costs are identified, and whether differences are found between young and older cohorts.

#### Findings for switch costs grouped by base-tasks

In the 85 studies reviewed, base-tasks varied in frequency of use. Digit base-tasks were most common, and were associated with the frequent reporting of both significant local-switch and global-switch costs. The prevalence of digit base-tasks may reflect their early use in task-switching research, as the first task-switching paradigm study by Jersild (1927) used a digit-based task. Independent of their prevalence, digit base-tasks had the highest mean effect sizes for local-switch costs measured by both RT and accuracy, as well as for global-switch costs measured by RT, compared with other base-task categories. Digit base-tasks have the desirable property of using a relatively small and standardised set of stimuli, such as the digits 0–9. This standardisation may reduce variation in stimulus characteristics across trials compared with paradigms in which the stimuli vary in features such as lexical content, or colour. However, the current evidence is not sufficient to inform us of whether the dominance of digit base-tasks is due to its widely accepted optimality in task-switching paradigm design, the need to replicate prior research, or a reflection of methodological inertia. This consideration echoes Bush et al.'s (2018) discussion of new and revised tests under the context of psychological and neuropsychological instruments.

The joint-second most frequently used base-task category were the colour and shape base-tasks and the colour and letter base-tasks. Colour and shape base-tasks are similar to the Wisconsin Card Sorting test (WCST, Berg, 1948)^4^, but the task-switching paradigm is a more direct measure of task-switching functioning than the WCST. Colour and letter base-tasks are similar to the Stroop task (Stroop, 1992)^5^, as both apply the tasks of word-reading and colour-naming. When the Stroop task was introduced into the task-switching setting as one of the base-tasks, the effort required for inhibition of the old task and activation of the new task was found to be asymmetrical: switching from the colour-naming task to the word-reading task was more effortful than switching from the word-reading task to the colour-naming task because the dominant word-reading task took more effort to inhibit (Allport et al., 1994). This asymmetry may be why we found that studies using colour and letter base-tasks had the lowest mean effect sizes on local-switch costs (as measured by RT and accuracy) and global-switch costs (as measured by RT). The different inhibition requirements between colour and letter tasks might play a role in the notably lower sensitivity of colour and letter base-tasks to switch-cost effects in the task-switching paradigm. Sensitivity here refers to the extent to which a given base-task pairing produces a detectable switch cost across studies, rather than implying that switch costs index a single, unitary switching ability. Task-switching performance is itself thought to draw on multiple separable processes, such as task-set reconfiguration, interference resolution, and cue encoding (Kiesel et al., 2010). No single task is likely to provide a pure measure of any one such process, since performance on any task necessarily reflects task-specific, non-switching processes alongside it (i.e., the task-impurity problem mentioned in Miyake et al., 2000).

#### Findings for switch costs grouped by switch-rules

The most frequently utilised switch-rule was cueing before each trial of the task with word cues. Studies utilising word-cued switch-rules had relatively lower effect sizes for local-switch costs and for global-switch costs. Consequently, using the task-switching paradigm with word-cued switch-rules might result in obtaining smaller but clearer switch costs, compared to task-switching paradigms utilising other switch-rules. This might be because the pre-acquired semantic meaning of word cues is more informative, and thus required less learning compared with other cues. This comports with Gaál and Czigler (2015 [Study 136]) who compared the magnitude of switch costs, and found more informative cues facilitated easier switching between tasks. This interpretation on cue informativeness was consistent with the findings of Nessler et al. (2012), where an informative cue led to decreased switch costs, compared to a non-informative cue. Beyond the context of task-switching, Fujawa and Strybel's (1997) findings also align with the effect of cue informativeness, a more informative auditory cue facilitates visual search, compared to a less informative auditory cue.

The next most frequently utilised switch-rule was cueing before each trial of the task with colour cues, followed by other visual cues. Compared to other switch-rules, studies utilising colour-cued switch-rules had relatively larger variations in effect sizes on local- and global-switch costs measured by RT. The effect sizes on global-switch costs measured by accuracy were relatively more consistent. Applying the same logic as above, this larger variation in effect sizes could result from the effect of lack of informativeness in the cue. Referring to the findings from Gaál and Czigler (2015 [Study 136]), word cues (i.e., more informative cues) reduced switch costs more than visual cues (i.e., less informative cues). The association between tasks and colour cues or the association between tasks and visual cues is more arbitrary than the association between tasks and word cues. This interpretation shows similarities with Jiménez et al.'s (2021) discussion on the effect of cues in Stroop-based tasks. When there was an arbitrary association between the stimuli and the responses, the cue was less effective due to higher cognitive load created by the task design. In task-switching paradigms, higher cognitive load can be created by utilising arbitrary associations between the task and the cue, which introduces greater variability in the sensitivity of colour-cued switch-rules in measuring task-switching performance.

Studies utilising visually-cued switch-rules (e.g., shape- or pattern-based cues) had larger effect sizes for local-switch costs measured by RT compared with studies utilising switch-rules from other categories (e.g., word-cued or colour-cued switch-rules). In contrast, there was a relatively smaller effect size for local-switch costs (as measured by accuracy) when comparing between studies utilising visually-cued switch-rules and studies utilising other switch-rule categories. One possible explanation for the discrepancy between effect sizes based on RT and accuracy is the informativeness of the cues used to signal task switches (Gaál & Czigler, 2015 [Study 136]; Nessler et al., 2012). However, the available evidence does not allow us to determine whether differences in cue informativeness contributed to the observed discrepancy between RT- and accuracy-based effect sizes.

### Switch costs—other paradigm manipulations

Complexity of the stimulus-features, such as bivalent stimulus *versus* univalent stimulus, was related to longer RT and greater local-switch costs (e.g., as in Ging-Jehli & Ratcliff, 2020; Hirsch et al., 2016; Lien et al., 2008; Rey-Mermet & Meier, 2015 [Study 489, 128, 127, 66]). Ging-Jehli and Ratcliff (2020) found complex stimulus-features were not only related to increased RT, but also to larger RT and accuracy differences between no-switch trials and switch trials, where the switch trials were subsequently slower and less accurate.

Complexity of the task-environment was also explored in the selected studies, with more complex and less informative task-environments being associated with larger switch costs (Gaál & Czigler, 2015; Hahn et al., 2004; Lien et al., 2008; Mayr, 2001; Van Asselen & Ridderinkhof, 2000; West & Travers, 2008 [Study 136, 89, 127, 82, 1036, 409]). This aligns with cognitive load theory, which states that higher cognitive load created by complexity of the material can lead to worse performance (Paas & van Merriënboer, 2020; Sweller et al., 2019).

Longer preparation-times (of all kinds) were associated with lower switch costs (Hirsch et al., 2016; Kramer et al., 1999; Kunimi et al., 2016; Lawo et al., 2012; Meiran et al., 2001 [Study 128, 1100, 732, 1278, 882]). One interpretation is that there was an enhancement in performance introduced by having more time in advance to prepare for the task-switching. This could be related to the potential explanation underlying the RT-accuracy trade-off found in older adults when responding to task-switching tasks (Van Asselen & Ridderinkhof, 2000), where a longer time was actively taken to prepare for responding to the task accurately. This effect of advance preparation is well-established in task-switching performance, and it is not restricted to only older populations (Altmann, 2004; Karayanidis et al., 2010). This effect of advance preparation can also apply to other aspects of executive functioning, such as working memory (e.g., Manelis & Reder, 2015).

Digit base-tasks were found to be the most used base-task with the largest reported effect size, among all variations of task-switching paradigms. This is partly due to using the same set of stimuli (i.e., digits 1, 2, 3, 4, 6, 7, 8, 9) across the two distinct base-tasks (i.e., digit-parity task and digit-comparison task). These stimuli had bivalent characteristics, which increased the difficulty of switching between tasks due to the greater level of interference between the two base-tasks (Ging-Jehli & Ratcliff, 2020). This effect of bivalent stimuli is aligned with “bivalency cost” discussed in Woodward et al. (2003) and with “bivalency effect” discussed in Grundy et al. (2013). Meier et al. (2015) also found the bivalency effect by testing task-switching performance in grapheme-colour synesthetes. The bivalent feature was created on univalent stimuli by introducing synesthetic colour experiences, which was associated with longer RTs in responding to tasks in the task-switching paradigm (Meier et al., 2015).

### Study demographics: sample size, age, gender, and publication bias

There is a constant debate in the field of experimental psychology in regards to under-powered studies due to smaller than required sample sizes (e.g., Lakens, 2022; Marszalek et al., 2011). In the 85 studies, the average total sample size was 67 participants, with 16 being the smallest number of participants and 369 being the largest. Using the sample size evaluation technique developed by Garner et al. (2022), we found evidence that statistically significant switch costs were not simple artefacts of large sample sizes. The findings of the current systematic review could help inform sample-size considerations for experimental studies in cognitive psychology. In task-switching research, some studies with samples of approximately 40–60 participants detected statistically significant effects, although the selection of appropriate sample size should depend on the expected effect size, study design, and statistical power.

There were large inconsistencies in how age was reported. Some studies did not report age range, while others reported age range but did not report means, standard deviations or age means. Precise age characteristics are an important basis for interpreting age-related performance, and thus reporting age and relevant measures of central tendency (e.g., mean and median) and variability (e.g., standard deviation) are critical for improving understanding of ageing trajectories. Cognitive function in ageing generally follows a pattern of gradual decline, then a change point followed by more rapid decline. Estimates from longitudinal research on cognitive functions suggests significant age-related decline occurred across multiple cognitive functions such as memory, attention and executive functions, and including task-switching, between 64.1 and 69.7 years (McCarrey et al., 2016). Longitudinal research suggests change points typically take place between 55 and 65, and this varies on the basis of the specific test, with an estimated 58 years of age for switching measured by Trail Making, and 62 years of age when measured by the WCST (Caselli et al., 2020). Future studies could consider precise reporting of the participants age range, mean and standard deviation to permit better comparison and generalisation across studies, and to capture the change point specific to the task-switching function. With this information available, future systematic reviews would be able to compare task-switching in young *versus* older adults across paradigms and studies.

Of the studies reviewed (68 out of 85), 80% reported gender characteristics and, of those, there was a bias toward including more women than men. This may be important as different ageing trajectories have been found in cognitive functioning between men and women. Compared to men in the same age group, women commonly have both better performance and greater resilience in multiple cognitive functions, including perceptuomotor speed (McCarrey et al., 2016) and episodic memory (Wahlin et al., 2006). Conversely, men typically outperform women on both visuospatial abilities (McCarrey et al., 2016) and semantic memory (Wahlin et al., 2006). However, the evidence supports no gender-related differences in task-switching performance (Hirsch et al., 2019), and in task-switching as assessed by Trail Making (Caselli et al., 2020; McCarrey et al., 2016). These findings suggest that generalising findings of the current review to both women and men is likely not a concern.

The level of detail in reporting the results varied across studies, with notable under-reporting of non-statistically significant results. It was common that the published studies found at least one statistically significant result, yet the reporting of other non-significant results was often overlooked. This suggests that studies that had no significant results failed to reach publication. Though we do not have sufficient evidence to comment on this directly, this aligns with publication and reporting bias that is an ongoing discussion in cognitive science (Ioannidis et al., 2014). A recent meta-meta-analysis suggested that the publication bias is still prevalent in industrial/organisational psychology, with bias assessment methods being underused, allowing for publication bias to be largely undetected (Siegel et al., 2022). The phenomenon of publication bias was also noted in the research on cognitive advantage introduced by bilingual experiences (de Bruin et al. 2015b), where authors stated that regardless of sample sizes and analysis methods, studies supporting bilingual advantage were more likely to be published, compared to studies against. In the studies reported in the current systematic review, non-significant results were either not reported, or consistently reported with minimal details, with many only reporting “F < 1” for non-significant ANOVA tests. Accordingly, in future studies, statistics relating to non-significant test results could be reported with the same level of detail as significant tests to avoid the potential impacts of bias. The necessity of reporting non-significant results was also discussed and embraced by the *Neurology* Null Hypothesis Special Edition (Petty & Gross, 2019), to encourage researchers to share non-significant results. Open Science Framework (Foster & Deardorff, 2017) has also emerged to support the scientific significance of non-significant results by encouraging pre-registration and open access of study methods.

### Strengths, limitations and future directions

The current review used a systematic approach to provide a comprehensive overview of task-switching performance in healthy older adults. This review built on previous findings (Kiesel et al., 2010; Wasylyshyn et al., 2011), aiming to capture how the task-switching paradigm has evolved over time, and explore the consequences this may have for research comparing task-switching performance across young and older adults. This required reviewing the details of paradigm manipulations, with findings related to task-switching performance being compared across different manipulations to understand the differential influence of these manipulations. The current review also paid attention to the use of different switch costs, with the outcomes of different switch costs being summarised and compared. As a result of this comprehensive review of manipulations to the task-switching paradigm and choice of different switch costs, the findings can be used to inform future studies about how design and selection of switch costs changed over time and how this influences task-switching performance.

The chosen databases for this review were carefully selected, but they were still not perfectly comprehensive. The four databases selected provide among the most comprehensive collections of psychological literature available (Paperpile, 2021), but may not have captured all of the relevant literature. In addition, this review was limited to English language only, meaning that the findings cannot be generalised to the findings of task-switching studies reported in languages other than English. As a result, relevant task-switching studies published in languages other than English may not have been identified, potentially limiting the cultural and linguistic diversity of the evidence represented in this review and restricting its applicability. For instance, based on a quick search in Baidu Scholar (Chinese equivalent to Google Scholar, searched in 2025) using the combined search terms “任务转换范式” (i.e., task-switching paradigm) and “老” (i.e., old, which is a character used in phrases such as older adult, older age, and later-life), 110 relevant results were found that were not considered in the current search due to the language inclusion criteria.

Studies written in languages other than English might provide a different view from the current group of studies reviewed, due to the influence of culture differences (e.g., language, education, mode of thinking) on cognitive functioning. As an example, Gul and Humphreys (2014) found that when participants were asked to switch between identifying the gender or the emotion of a face, participants from Britain were faster at identifying gender, whereas participants from Pakistan were faster at identifying emotions. The authors suggested that this is because different culture groups were focused on different characteristics of a face. Another finding of the study was that participants from Pakistan performed task-switching with smaller switch costs than participants from Britain, possibly due to the effect of bilingualism (Gul & Humphreys, 2014). Experiments conducted by Prior and Gollan (2011) suggested that the language-switching behaviour that was commonly experienced by bilinguals was associated with better task-switching performance. The age-related decline in task-switching was also reduced in lifelong bilinguals, compared to monolinguals (Houtzager et al., 2017). Therefore, it is likely that paradigm manipulations could have diverse effects across different culture groups with different language backgrounds. The cross-culture comparison can be a topic of future reviews in the investigation of task-switching function.

The current systematic review focused only on task-switching in the context of comparing healthy young and older adults. Although the variety of paradigms aligns with evidence that cognitive tests in general have evolved and become more varied over time (McCarrey et al., 2016), it is unclear if findings here are applicable to task-switching performance across all ages, health conditions, and task-switching paradigms that are not included in the current review. Future reviews could examine paradigm manipulations and their effect on task-switching performance across all ages, or in samples such as older adults with cognitive impairments. This would facilitate greater understanding of the specificity and generalisability of the findings of the current review to further the understanding of task-switching idiosyncrasies.

### Conclusion

The current systematic review provides an overview of the effects on task-switching performance of the different manipulations that have been introduced to the task-switching paradigm. The most common base-task was the digit base-task, and the most common switch-rule was the word-cued switch-rule. The most common measure of switch costs was the local-switch cost measured by RT.

Overall, switch costs were frequently reported across multiple base-task and switch-rule categories and in studies involving both young and older participants. Substantial variability was observed in how task-switching was measured and how both demographic and substantive results were reported. This makes it difficult to determine whether the apparent consistency of switch-cost findings across paradigms reflects a genuine pattern or under-reporting of null results.

Taken together, the findings indicate that digit base-tasks, word-cued switch-rules, and local-switch costs were among the most commonly reported paradigm features and outcome measures in the reviewed literature. However, due to variability in reporting across studies, the limited availability of effect-size information, and the lack of direct comparisons between different base-tasks and switch-rules, these findings should be interpreted with caution.

Future research with greater consistency in reporting could confirm and build on the current findings, facilitate comparisons between different manipulations of task-switching paradigms, and clarify whether paradigm manipulations affect the ability of task-switching paradigms to capture task-switching function in healthy young and older adults.

## Supplementary Information

Below is the link to the electronic supplementary material.

Supplementary Material 1 (DOCX 361 KB)

## Data Availability

No datasets were generated or analysed during the current study.

## Footnotes

1 Where explicit age information was unavailable in the title and/or abstract, journal articles were retained for investigating as part of the full-text screening. Articles were included if the age group had substantial overlap with age < 60 years (e.g., age range from 50 to 60 years), or used terms associated with this age group (e.g., old-old, elderly, etc.).

2 Note that common risk factors that may potentially lead to physical brain damage, such as alcohol consumption and smoking, were not used as the basis for exclusion given their high prevalence in the broader population, and lower likelihood of being reported in studies focusing on general population samples.

3 It is important to state that there was a notable under-reporting of detail for non-significant results. Where reported, non-significant switch costs were found in studies with the colour and shape task category, the colour and letter task category, the switch between three tasks category, and the semantic and non-semantic task category. (Further details for each base-task category can be found in the Supplementary Materials Table 3.) Non-significant switch costs were found across most of the switch cost types, with the exception of global-switch costs measured by reaction time (RT).

4 The WCST is also a common measure of task-switching ability that incorporates the tasks of colour-discrimination and shape-discrimination. However, the WCST requires participants to categorise the stimulus, and the rules of both categorisation and task-switching are not directly presented to the participants. This makes performance on the WCST depend not only on the task-switching function, but also on other functions such as decision-making.

5 For the Stroop task, the colour words (e.g., Blue, Green) are printed in various font colours (e.g., Yellow, Green). The “colour words” and the “font colours” could be either consistent or inconsistent. The participants’ task was either a word-reading task (i.e., read the colour word; stimulus: , answer: Green), or a colour-naming task (i.e., name the font colour; stimulus: , answer: Blue). From the perspective of an inhibitory account, when these two tasks conflict with each other, inhibiting the intention to perform the word-reading task was harder than inhibiting the intention to perform the colour-naming task.
