# Supplementary material for: A systematic review of task-switching in healthy older adults: paradigm manipulations and switch costs
Source: Psychol Res. 2026 Nov 12;90(6):208. doi: 10.1007/s00426-026-02398-z (PMC13759586; doi:10.1007/s00426-026-02398-z)
Supplement: Supplementary file 1 [file 426_2026_2398_MOESM1_ESM.docx]

**A Systematic Review of Task-Switching in Healthy Older Adults: Paradigm Manipulations and Switch Costs**

**Supplementary Materials**

**A Submission to *Psychological Research***

**Authers Names and Affiliations**

Xuanning He^a^, Erin I. Walsh^a^, and Anne M. Aimola Davies^a^,

^a^The Australian National University, Canberra, ACT, Australia

**Corresponding Author**

Xuanning He

Email: [Xuanning.He@anu.edu.au](mailto:Xuanning.He@anu.edu.au)

Address: Building 39, Science Rd, The Australia National University, Canberra, ACT, Australia, 2601.

**Supplementary Materials**

**Table of Content:**

1. **Supplementary Table 1. Summary of Information Extracted………………. Page 3**
2. **Supplementary Table 2. References for Studies Reviewed in**

**The Systematic Review…………..………………………………………………Page 10**

3. **Supplementary Figure 1. Publication Year for the Records (N = 73) ………..Page 20**
4. **Supplementary Table 3. Summary of Base-Tasks………………..…...……….Page 21**
5. **Supplementary Table 4. Effect Sizes of Local-Switch Costs (Reaction Time): Descriptive Summary………………………………………..……….………...Page 26**
6. **Supplementary Table 5. Effect Sizes of Local-Switch Costs (Accuracy):**

**Descriptive Summary……………….……………….…………….…………….Page 27**

7. **Supplementary Table 6. Effect Sizes of Global-Switch Costs (Reaction Time): Descriptive Summary………………………...……………..………….………..Page 28**
8. **Supplementary Table 7. Effect Sizes of Global-Switch Costs (Accuracy): Descriptive Summary………………………...………………..……..…...……..Page 29**
9. **Supplementary Figure 2. Effect Sizes of Local-Switch
   Costs (Reaction Time)…………………………………………………..…….....Page 30**
10. **Supplementary Figure 3. Effect Sizes of Local-Switch
    Costs (Accuracy)…………………………………………………..…......………Page 31**
11. **Supplementary Figure 4. Effect Sizes of Global-Switch
    Costs (Reaction Time)……………………………………………...……..……..Page 32**
12. **Supplementary Figure 5. Effect Sizes of Global-Switch
    Costs (Accuracy)…………………………………………………..…....…..……Page 33**
13. **Supplementary Materials: Code for Calculating Partial Eta-Squared………………………………………………..…………..……...………Page 34**
14. **Supplementary Materials: Descriptive Assessment of the Combinations of Base-Tasks and Switch-Rules…………………………………………………………Page 35**

| Supplementary Table 1  *Summary of Information Extracted* | | | |
| --- | --- | --- | --- |
| Variable | Note | Form |  |
| Basic Journal Article Information | |  | |
| Place of Study | In which country did the study take place? | Categorical | |
| Included Studies | Number of sub-studies included in the selected articles. (Each study was reported on a new row of the excel extraction sheet.) | Numeric | |
| Study Number | Ordinal study number of the reported study for the current row (i.e., study number 1 out of 3 included studies identified within a given included journal article). | Numeric | |
| Sample Size | Total number of participants. | Numeric | |
| Older Adult Sample Size | If the study was included because the sub-sample fulfilled the inclusion criteria, we extracted the sub-sample size of older adults (e.g., 20 older adults out of a total sample of 40 participants). | Numeric | |
| Comparison Group | Brief explanation of the characteristics of the comparison group. | String | |
| Comparison Group Sample Size | Number of participants in the comparison group. Each comparison group had its own column. | Numeric | |
| Multi-lingual Participants | Whether participants reported in the study spoke more than one language (0 = no, 1 = yes). | Boolean | |
| Language Spoken | A list of languages spoken by participants. When information was available, languages were listed from first language to second language. | String | |
| Basic Journal Article Information—Continued | | | |
| Age Mean | Mean age of the participants. (Older group, and each comparison group had their own column.) | Numeric | |
| Age SD | The standard deviation of the mean age. (Older group, and each comparison group had their own column.) | Numeric | |
| Age Minimum | Youngest participant’s age. (Older group, and each comparison group had their own column.) | Numeric | |
| Age Maximum | Oldest participant’s age. (Older group, and each comparison group had their own column.) | Numeric | |
| Gender (Number of Women) | Number of women participants. (Older group, and each comparison group had their own column.) | Numeric | |
| Experimental Paradigm Information | |  | |
| Type of Task-Switching Base-Tasks | Description of the type of tasks participants were required to switch between (i.e., description of characteristics of a single trial; e.g., shape-discrimination task). | String | |
| Type of Task Switch-Rules | Description of how switch behaviour was introduced to participants (e.g., switching voluntarily, memorising the switch-rules, and cueing before the task, etc.). | String | |
| Number of Pure Blocks | Number of pure blocks each participant completed. | Numeric | |
| Number of Trials (Pure) | Number of pure test trials each participant completed. | Numeric | |
| Number of Mixed Blocks | Number of mixed blocks each participant completed. | Numeric | |
| Number of Trials (Mixed) | Number of mixed test trials each participant completed. | Numeric | |
| Number of Practice Blocks | Number of practice blocks each participant completed. | Numeric | |
| Number of Trials (Practice) | Number of practice trials each participant completed. | Numeric | |
| Stimulus Presentation Time | Time Duration of stimulus presented on the screen. If this varied, used the format: Varied (list of different times). | Numeric  (with unit) | |
| Preparation Time Between Trials | Time Duration between each trial. If this varied, used the format: Varied (list of different times). | Numeric  (with unit) | |
|  |  |  | |
| Switch Costs Information |  |  | |
| Number of Switch Costs | Number of different types of switch-costs reported in the article (e.g., local-switch costs or global-switch costs). | Numeric | |
| Names of Switch Cost | Names of reported switch cost. Each switch cost had their own row. (e.g., RT Global-Switch Cost). | String | |
| Switch Analysis Type | Statistical tests used to analyse the switch performance (e.g., ANOVA). | String | |
| Switch Mean | Performance mean for switch trials. | Numeric | |
| Switch SD | Performance SD for switch trials. | Numeric | |
| No-Switch Mean | Performance mean for no-switch trials. | Numeric | |
| No-Switch SD | Performance SD for no-switch trials. | Numeric | |
| Mean Switch-Cost | Performance mean for switch-costs. | Numeric | |
| SD Switch-Cost | Performance SD for switch-costs. | Numeric | |
| Switch Significance | The reported significance of the switch-cost statistical tests (0 = non-significant, 1 = significant). | Boolean | |
| Switch Analysis Results | The detailed results of the analysis. If the exact number (e.g., *F*-statistics) was not reported in the article, provided a short phrase or sentence that described the result (e.g., *F*<1). | String | |
| Error Trials Included | If the measure was in RT, were error trials included in the RT calculations (Yes, No, or Unspecified). | Categorical | |
| Additional Relevant Information (e.g., factors related to task-switching, but not manipulated by the paradigm) | |  | |
| Other Related Factors | Number of explicit investigations of other factors related to task-switching. If an article included multiple studies, each study should be calculated independently, and have their own row. | Count | |
| Other Related Factors: Category | Category of factors that were suggested to be related to task-switching. Categories included:   - Age - Variations on task-switching paradigm - Executive function or Cognitive function - Neurological - Lifestyle - Other   Interactions between multiple variables were listed separately from the main effect, using the category "Interaction". | Categorical | |
| Other Factors: Name | The name of the factor related to task-switching. Each factor had their own row. | String | |
| Measurement Category of Other Factors | Category of measurement used to represent other factors (e.g., objective measure, self-report, validated questionnaire). | String | |
| Measurement Name | The name of the tool or questionnaire used to represent the identified factor. | String | |
| Key Hypothesis | Report of the following information of the key hypothesis related to factors other than the paradigm itself:   - Conceptual relationship with task-switching proposed in the article (i.e., hypothesis and justification for making this hypothesis). - Whether the key hypothesis was supported. - Statistical analysis used to analyse the key hypothesis. - The result of the statistical analysis. If the exact number (e.g., *F*-statistics) was not reported in the article, provided a sentence that described the results (e.g., *F*<1). | String | |
| Mediator/Moderator | Report of the following information of the mediator/moderator related to factors other than the paradigm itself:   - Was a mediator or moderator mentioned in the hypothesis. - Was the effect of the mediator or moderator supported. Briefly explain. - Statistical analysis used to analyse the effect of the mediator or moderator. - The result of the statistical analysis. If the exact number (e.g., *F*-statistics) was not reported in the article, provided a sentence that described the results (e.g., *F*<1). | String | |
| Longitudinal Design Information | |  | |
| Longitudinal Design | Was the study a longitudinal design (0 = no, 1 = yes). | Boolean | |
| Longitudinal Hypothesis | Conceptual explanation or expectation about longitudinal change over time. | String | |
| Number of Time Points | Number of longitudinal time points for each participant. | Numeric | |
| Time Duration Between Data Collection | Time duration between the first and last point of data collection. | Numeric (time unit) | |
| Longitudinal Relationship | The result of the statistical analysis on the longitudinal change overtime. If the exact number (e.g., *F*-statistics) was not reported in the article, provided a sentence that described the results (e.g., *F*<1). | Numeric /string | |
| Task-Switching Definition Information | |  | |
| Definition of Task-Switching | If the study included a definition of task-switching, report the definition. | String | |
| Task-Switching Concept | The authors' suggestions on what the task-switching paradigm was measuring. | String | |
| Source of Switch Cost | The suggested theoretical source of switch-costs. | String | |
| Risk of Bias Information | |  | |
| Risk of Bias | Based on the questions provided by (Rosella et al., 2016, <https://ars.els-cdn.com/content/image/1-s2.0-S0033350615004370-mmc1.pdf>), was there a risk of bias (No = 0, Yes = 1). | Boolean | |
| Note for the Bias | Explain potential risk of bias. | String | |
| *Note.* “Variable” column is a description of the information. “Note” column contains a detailed description of the expected variables. “Forms” column is an indication of the expected variable form of the extracted information. | | | |

| **Supplementary Table 2**  *References for Studies Reviewed in the Systematic Review* | |
| --- | --- |
| **Study Number** | **Reference** |
| Study 30 | Mulligan, B. P., Segalowitz, S. J., Hofer, S. M., & Smart, C. M. (2020). A multi-timescale, multi-method perspective on older adult neurocognitive adaptability. *The Clinical Neuropsychologist, 34*(4), 643–677. https://doi.org/10.1080/13854046.2020.1723706 |
| Study 53 | West, R., & Moore, K. (2005). Adjustments of Cognitive Control in Younger and Older Adults. *Cortex, 41*(4), 570–581. https://doi.org/10.1016/S0010-9452(08)70197-7 |
| Study 56 | Madden, D. J., Costello, M. C., Dennis, N. A., Davis, S. W., Shepler, A. M., Spaniol, J., Bucur, B., & Cabeza, R. (2010). Adult age differences in functional connectivity during executive ontrol. *NeuroImage*, *52*(2), 643–657. https://doi.org/10.1016/j.neuroimage.2010.04.249 |
| Study 66 | Rey-Mermet, A., & Meier, B. (2015). Age-related effects over bilingual language control and executive control. *Aging, Neuropsychology, and Cognition, 22*(1), 72–94. https://doi.org/10.1080/13825585.2014.889070 |
| Study 67 | Themanson, J. R., Hillman, C. H., & Curtin, J. J. (2006). Age and physical activity influences on action monitoring during task switching. *Neurobiology of Aging, 27*(9), 1335–1345. https://doi.org/10.1016/j.neurobiolaging.2005.07.002 |
| Study 70 | Kray, J., Eppinger, B., & Mecklinger, A. (2005). Age differences in attentional control: An event-related potential approach. *Psychophysiology, 42*(4), 407–416. https://doi.org/10.1111/j.1469-8986.2005.00298.x |
| Study 78 | Eppinger, B., Kray, J., Mecklinger, A., & John, O. (2007). Age differences in task switching and response monitoring: Evidence from ERPs. *Biological Psychology, 75*(1), 52–67. https://doi.org/10.1016/j.biopsycho.2006.12.001 |
| Study 82 | Mayr, U. (2001). Age differences in the selection of mental sets: The role of inhibition, stimulus ambiguity, and response-set overlap. *Psychology and Aging, 16*(1), 96–109. https://doi.org/10.1037/0882-7974.16.1.96 |
| Study 85 | Butler, K. M., & Weywadt, C. (2013). Age differences in voluntary task switching. *Psychology and Aging, 28*(4), 1024–1031. https://doi.org/10.1037/a0034937 |
| Study 86 | Gazes, Y., Rakitin, B. C., Habeck, C., Steffener, J., & Stern, Y. (2012). Age differences of multivariate network expressions during task-switching and their associations with behavior. *Neuropsychologia, 50*(14), 3509–3518. https://doi.org/10.1016/j.neuropsychologia.2012.09.039 |
| Study 88 | Bojko, A., Kramer, A. F., & Peterson, M. S. (2004). Age Equivalence in Switch Costs for Prosaccade and Antisaccade Tasks. *Psychology and Aging, 19*(1), 226–234. https://doi.org/10.1037/0882-7974.19.1.226 |
| Study 89 | Hahn, S., Andersen, G. J., & Kramer, A. F. (2004). Age Influences on Multi-Dimensional Set Switching. *Aging, Neuropsychology, and Cognition, 11*(1), 25–36. https://doi.org/10.1076/anec.11.1.25.29360 |
| Study 95 | Eich, T. S., MacKay-Brandt, A., Stern, Y., & Gopher, D. (2016). Age-Based Differences in Task Switching Are Moderated by Executive Control Demands. *The Journals of Gerontology Series B: Psychological Sciences and Social Sciences*, gbw117. https://doi.org/10.1093/geronb/gbw117 |
| Study 110 | Friedman, D., Nessler, D., Johnson, R., Ritter, W., & Bersick, M. (2007). Age-Related Changes in Executive Function: An Event-Related Potential (ERP) Investigation of Task-Switching. Aging, *Neuropsychology, and Cognition, 15*(1), 95–128. https://doi.org/10.1080/13825580701533769 |
| Study 111 | Kopp, B., Lange, F., Howe, J., & Wessel, K. (2014). Age-related changes in neural recruitment for cognitive control. *Brain and Cognition, 85*, 209–219. https://doi.org/10.1016/j.bandc.2013.12.008 |
| Study 121 | Chang, W. P., Shen, I. H., Wen, C.P., & Chen, C. L. (2020). Age-Related Differences Between Young and Old Adults: Effects of Advance Information on Task Switching. *Perceptual and Motor Skills, 127*(6), 985–1014. https://doi.org/10.1177/0031512520930872 |
| Study 127 | Lien, M. C., Ruthruff, E., & Kuhns, D. (2008). Age-related differences in switching between cognitive tasks: Does internal control ability decline with age? *Psychology and Aging, 23*(2), 330–341. https://doi.org/10.1037/0882-7974.23.2.330 |
| Study 128 | Hirsch, P., Schwarzkopp, T., Declerck, M., Reese, S., & Koch, I. (2016). Age-related differences in task switching and task preparation: Exploring the role of task-set competition. *Acta Psychologica, 170*, 66–73. https://doi.org/10.1016/j.actpsy.2016.06.008 |
| Study 132 | Calabria, M., Branzi, F. M., Marne, P., Hernández, M., & Costa, A. (2015). Age-related effects over bilingual language control and executive control. *Bilingualism: Language and Cognition, 18*(1), 65–78. https://doi.org/10.1017/S1366728913000138 |
| Study 134 | Zhu, Z., Hakun, J. G., Johnson, N. F., & Gold, B. T. (2014). Age-related increases in right frontal activation during task switching are mediated by reaction time and white matter microstructure. *Neuroscience*, 278, 51–61. https://doi.org/10.1016/j.neuroscience.2014.07.076 |
| Study 136 | Gaál, Z. A., & Czigler, I. (2015). Age-related processing strategies and go–nogo effects in task-switching: An ERP study. *Frontiers in Human Neuroscience,* 09. https://doi.org/10.3389/fnhum.2015.00177 |
| Study 137 | Jimura, K., & Braver, T. S. (2010). Age-Related Shifts in Brain Activity Dynamics during Task Switching. *Cerebral Cortex, 20*(6), 1420–1431. https://doi.org/10.1093/cercor/bhp206 |
| Study 138 | Gold, B. T., Powell, D. K., Xuan, L., Jicha, G. A., & Smith, C. D. (2010). Age-related slowing of task switching is associated with decreased integrity of frontoparietal white matter. *Neurobiology of Aging, 31*(3), 512–522. https://doi.org/10.1016/j.neurobiolaging.2008.04.005 |
| Study 143 | Hahn, S., Kramer, A. F., & Gopher, D. (1997). Aging and Executive Control in Task Switching. *Proceedings of the Human Factors and Ergonomics Society Annual Meeting*, 41(1), 105–109. https://doi.org/10.1177/107118139704100125 |
| Study 149\ | Terry, C. P., & Sliwinski, M. J. (2012). Aging and Random Task Switching: The Role of Endogenous Versus Exogenous Task Selection. *Experimental Aging Research, 38*(1), 87–109. https://doi.org/10.1080/0361073X.2012.637008 |
| Study 162 | Verhaeghen, P., & Hoyer, W. J. (2007). Aging, Focus Switching, and Task Switching in a Continuous Calculation Task: Evidence Toward a New Working Memory Control Process. *Aging, Neuropsychology, and Cognition, 14*(1), 22–39. https://doi.org/10.1080/138255890969357 |
| Study 165 | Serbruyns, L., Leunissen, I., van Ruitenbeek, P., Pauwels, L., Caeyenberghs, K., Solesio-Jofre, E., Geurts, M., Cuypers, K., Meesen, R. L., Sunaert, S., Leemans, A., & Swinnen, S. P. (2016). Alterations in brain white matter contributing to age-related slowing of task switching performance: The role of radial diffusivity and magnetization transfer ratio. *Human Brain Mapping, 37*(11), 4084–4098. https://doi.org/10.1002/hbm.23297 |
| Study 271 | Verstynen, T. D., Lynch, B., Miller, D. L., Voss, M. W., Prakash, R. S., Chaddock, L., Basak, C., Szabo, A., Olson, E. A., Wojcicki, T. R., Fanning, J., Gothe, N. P., McAuley, E., Kramer, A. F., & Erickson, K. I. (2012). Caudate Nucleus Volume Mediates the Link between Cardiorespiratory Fitness and Cognitive Flexibility in Older Adults. *Journal of Aging Research, 2012*, 1–11. https://doi.org/10.1155/2012/939285 |
| Study 272 | Madden, D. J., Spaniol, J., Costello, M. C., Bucur, B., White, L. E., Cabeza, R., Davis, S. W., Dennis, N. A., Provenzale, J. M., & Huettel, S. A. (2008). Cerebral White Matter Integrity Mediates Adult Age Differences in Cognitive Performance. *Journal of Cognitive Neuroscience, 21*(2), 289–302. https://doi.org/10.1162/jocn.2009.21047 |
| Study 341 | Mayr, U., & Kliegl, R. (2000). Complex semantic processing in old age: Does it stay or does it go? *Psychology and Aging, 15*(1), 29–43. https://doi.org/10.1037/0882-7974.15.1.29 |
| Study 391 | Wetter, S. R., Delis, D. C., Houston, W. S., Jacobson, M. W., Lansing, A., Cobell, K., Salmon, D. P., & Bondi, M. W. (2005). Deficits in Inhibition and Flexibility are Associated with the APOE-E4 Allele in Nondemented Older Adults. *Journal of Clinical and Experimental Neuropsychology, 27*(8), 943–952. https://doi.org/10.1080/13803390490919001 |
| Study 401 | Glisky, E. L., Alexander, G. E., Hou, M., Kawa, K., Woolverton, C. B., Zigman, E. K., Nguyen, L. A., Haws, K., Figueredo, A. J., & Ryan, L. (2021). Differences between young and older adults in unity and diversity of executive functions. *Aging, Neuropsychology, and Cognition, 28*(6), 829–854. https://doi.org/10.1080/13825585.2020.1830936 |
| Study 409 | West, R., & Travers, S. (2008). Differential effects of aging on processes underlying task switching. *Brain and Cognition, 68*(1), 67–80. https://doi.org/10.1016/j.bandc.2008.03.001 |
| Study 489 | Ging-Jehli, N. R., & Ratcliff, R. (2020). Effects of aging in a task-switch paradigm with the diffusion decision model. *Psychology and Aging, 35*(6), 850–865. https://doi.org/10.1037/pag0000562 |
| Study 494 | Kray, J., & Eppinger, B. (2006). Effects of associative learning on age differences in task-set switching. *Acta Psychologica, 123*(3), 187–203. https://doi.org/10.1016/j.actpsy.2005.12.009 |
| Study 540 | Hakun, J. G., Zhu, Z., Johnson, N. F., & Gold, B. T. (2015). Evidence for reduced efficiency and successful compensation in older adults during task switching. *Cortex, 64*, 352–362. https://doi.org/10.1016/j.cortex.2014.12.006 |
| Study 545 | de Bruin, A., Bak, T. H., & Della Sala, S. (2015). Examining the effects of active versus inactive bilingualism on executive control in a carefully matched non-immigrant sample. *Journal of Memory and Language, 85*, 15–26. https://doi.org/10.1016/j.jml.2015.07.001 |
| Study 554 | Vaughan, L., & Giovanello, K. (2010). Executive function in daily life: Age-related influences of executive processes on instrumental activities of daily living. *Psychology and Aging, 25*(2), 343–355. https://doi.org/10.1037/a0017729 |
| Study 568 | Dai, C.-T., Chang, Y.-K., Huang, C.-J., & Hung, T.-M. (2013). Exercise mode and executive function in older adults: An ERP study of task-switching. *Brain and Cognition, 83*(2), 153–162. https://doi.org/10.1016/j.bandc.2013.07.007 |
| Study 569 | Li, D., Huang, C.-J., Liu, S.-C., Chang, K.-H., & Hung, T.-M. (2019). Exercise type relates to inhibitory and error processing functions in older adults. *Aging, Neuropsychology, and Cognition, 26*(6), 865–881. https://doi.org/10.1080/13825585.2018.1540688 |
| Study 570 | Tsai, C.-L., & Wang, W.-L. (2015). Exercise-mode-related changes in task-switching performance in the elderly. *Frontiers in Behavioral Neuroscience*, 9. https://doi.org/10.3389/fnbeh.2015.00056 |
| Study 576 | Sombric, C. J., Harker, H. M., Sparto, P. J., & Torres-Oviedo, G. (2017). Explicit Action Switching Interferes with the Context-Specificity of Motor Memories in Older Adults. *Frontiers in Aging Neuroscience,* 9. https://doi.org/10.3389/fnagi.2017.00040 |
| Study 607 | Eich, T. S., Parker, D., Liu, D., Oh, H., Razlighi, Q., Gazes, Y., Habeck, C., & Stern, Y. (2016). Functional brain and age-related changes associated with congruency in task switching. *Neuropsychologia, 91*, 211–221. https://doi.org/10.1016/j.neuropsychologia.2016.08.009 |
| Study 612 | Gazes, Y., Habeck, C., O’Shea, D., Razlighi, Q. R., Steffener, J., & Stern, Y. (2015). Functional network mediates age‐related differences in reaction time: A replication and extension study. *Brain and Behavior, 5*(5). https://doi.org/10.1002/brb3.324 |
| Study 621 | DiGirolamo, G. J., Kramer, A. F., Barad, V., Cepeda, N. J., Weissman, D. H., Milham, M. P., Wszalek, T. M., Cohen, N. J., Banich, M. T., Webb, A., Belopolsky, A. V., & McAuley, E. (2001). General and task-specific frontal lobe recruitment in older adults during executive processes: A fMRI investigation of task-switching. *Neuroreport, 12*(9), 2065–2071. https://doi.org/10.1097/00001756-200107030-00054 |
| Study 627 | Erickson, K. I. (2008). Genetic contributions to age-related decline in executive function: A 10-year longitudinal study of COMT and BDNF polymorphisms. *Frontiers in Human Neuroscience*, 2. https://doi.org/10.3389/neuro.09.011.2008 |
| Study 694 | Qin, S., & Basak, C. (2020). Influence of Multiple Cardiovascular Risk Factors on Task-Switching in Older Adults: An fMRI Study. *Frontiers in Human Neuroscience*, 14, 561877. https://doi.org/10.3389/fnhum.2020.561877 |
| Study 732 | Kunimi, M., Kiyama, S., & Nakai, T. (2016). Investigation of age-related changes in brain activity during the divalent task-switching paradigm using functional MRI. *Neuroscience Research, 103*, 18–26. https://doi.org/10.1016/j.neures.2015.06.011 |
| Study 763 | Gajewski, P. D., & Falkenstein, M. (2015). Lifelong physical activity and executive functions in older age assessed by memory based task switching. *Neuropsychologia, 73*, 195–207. https://doi.org/10.1016/j.neuropsychologia.2015.04.031 |
| Study 833 | Enriquez-Geppert, S., & Barceló, F. (2018). Multisubject Decomposition of Event-related Positivities in Cognitive Control: Tackling Age-related Changes in Reactive Control. *Brain Topography, 31*(1), 17–34. https://doi.org/10.1007/s10548-016-0512-4 |
| Study 857 | Head, D., Rodrigue, K. M., Kennedy, K. M., & Raz, N. (2008). Neuroanatomical and cognitive mediators of age-related differences in episodic memory. *Neuropsychology, 22*(4), 491–507. https://doi.org/10.1037/0894-4105.22.4.491 |
| Study 861 | Steffener, J., Barulli, D., Habeck, C., & Stern, Y. (2014). Neuroimaging explanations of age-related differences in task performance. *Frontiers in Aging Neuroscience,* 6. https://doi.org/10.3389/fnagi.2014.00046 |
| Study 882 | Meiran, N., Gotler, A., & Perlman, A. (2001). Old Age Is Associated With a Pattern of Relatively Intact and Relatively Impaired Task-Set Switching Abilities. *The Journals of Gerontology Series B: Psychological Sciences and Social Sciences, 56*(2), P88–P102. https://doi.org/10.1093/geronb/56.2.P88 |
| Study 943 | Albinet, C. T., Boucard, G., Bouquet, C. A., & Audiffren, M. (2012). Processing speed and executive functions in cognitive aging: How to disentangle their mutual relationship? *Brain and Cognition, 79*(1), 1–11. https://doi.org/10.1016/j.bandc.2012.02.001 |
| Study 963 | Zhu, Z., Johnson, N. F., Kim, C., & Gold, B. T. (2015). Reduced Frontal Cortex Efficiency is Associated with Lower White Matter Integrity in Aging. *Cerebral Cortex, 25*(1), 138–146. https://doi.org/10.1093/cercor/bht212 |
| Study 1036 | Van Asselen, M., & Ridderinkhof, K. R. (2000). Shift Costs of Predictable and Unexpected Set Shifting in Young and Older Adults. *Psychologica Belgica, 40*(4), 259. https://doi.org/10.5334/pb.966 |
| Study 1100 | Kramer, A. F., Hahn, S., & Gopher, D. (1999). Task coordination and aging: Explorations of executive control processes in the task switching paradigm. *Acta Psychologica, 101*(2–3), 339–378. https://doi.org/10.1016/S0001-6918(99)00011-6 |
| Study 1140 | Fong, D. Y., Chi, L. K., Li, F., & Chang, Y. K. (2014). The benefits of endurance exercise and Tai Chi Chuan for the task-switching aspect of executive function in older adults: An ERP study. *Frontiers in Aging Neuroscience,* 6. https://doi.org/10.3389/fnagi.2014.00295 |
| Study 1211 | Steffener, J., Gazes, Y., Habeck, C., & Stern, Y. (2016). The Indirect Effect of Age Group on Switch Costs via Gray Matter Volume and Task-Related Brain Activity. *Frontiers in Aging Neuroscience*, 8. https://doi.org/10.3389/fnagi.2016.00162 |
| Study 1230 | Gajewski, P. D., Hengstler, J. G., Golka, K., Falkenstein, M., & Beste, C. (2011). The Met-allele of the BDNF Val66Met polymorphism enhances task switching in elderly. *Neurobiology of Aging, 32*(12), 2327.e7-2327.e19. https://doi.org/10.1016/j.neurobiolaging.2011.06.010 |
| Study 1267 | Simon, A., & Bock, O. (2017). The role of cognition for different stages of visuomotor adaptation in younger and older adults. *Human Movement Science, 52*, 215–222. https://doi.org/10.1016/j.humov.2017.02.011 |
| Study 1270 | Prakash, R. S., Hussain, M. A., & Schirda, B. (2015). The role of emotion regulation and cognitive control in the association between mindfulness disposition and stress. *Psychology and Aging, 30*(1), 160–171. https://doi.org/10.1037/a0038544 |
| Study 1278 | Lawo, V., Philipp, A. M., Schuch, S., & Koch, I. (2012). The role of task preparation and task inhibition in age-related task-switching deficits. *Psychology and Aging, 27*(4), 1130–1137. https://doi.org/10.1037/a0027455 |
| Study 1281 | Pettigrew, C., & Martin, R. C. (2016). The role of working memory capacity and interference resolution mechanisms in task switching. *Quarterly Journal of Experimental Psychology, 69*(12), 2431–2451. https://doi.org/10.1080/17470218.2015.1121282 |
| Study 1370 | Hawkes, T. D., Siu, K. C., Silsupadol, P., & Woollacott, M. H. (2012). Why does older adults’ balance become less stable when walking and performing a secondary task? Examination of attentional switching abilities. *Gait & Posture, 35*(1), 159–163. https://doi.org/10.1016/j.gaitpost.2011.09.001 |
| Study 1519 | Idowu, M. I., & Szameitat, A. J. (2023). Executive function abilities in cognitively healthy young and older adults—A cross-sectional study. *Frontiers in Aging Neuroscience*, *15*, 976915. <https://doi.org/10.3389/fnagi.2023.976915> |
| Study 1526 | Li, T., Xia, H., Li, H., He, Q., & Chen, A. (2023). Functional Connectivity Alterations of Cognitive Flexibility in Aging: Different Patterns of Global and Local Switch Costs. *The Journals of Gerontology: Series B*, gbad092. https://doi.org/10.1093/geronb/gbad092 |
| Study 1550 | Nagy, B., Czigler, I., Csizmadia, P., File, D., Fáy, N., & Gaál, Z. A. (2023). Investigating the involvement of cognitive control processes in innovative and adaptive creativity and their age-related changes. Frontiers in Human Neuroscience, 17, 1033508. https://doi.org/10.3389/fnhum.2023.1033508 |
| Study 1758 | Xia, H., Li, T., Hou, Y., Liu, Z., & Chen, A. (2024). Age-related decline in cognitive flexibility and inadequate preparation: evidence from task-state network analysis. GeroScience, 46(6), 5939–5953. https://doi.org/10.1007/s11357-024-01135-x |
| Study 1792 | Skolasinska, P., Qin, S., Voss, M., Lee, H. K., & Basak, C. (2024). Brain activations elicited during task‐switching generalize beyond the task: A partial least squares correlation approach to combine fMRI signals and cognition. Human Brain Mapping, 45(11), e26804-n/a. https://doi.org/10.1002/hbm.26804 |
| Study 1819 | Radović, T., Kübler, S., Mikolajczyk, R., Kluttig, A., Maydych, V., & Schubert, T. (2025). Cognitive flexibility in aging: the impact of age range and task difficulty on local switch costs in task switching. Frontiers in Aging Neuroscience, 17, 1619441. https://doi.org/10.3389/fnagi.2025.1619441 |
| Study 1968 | Wu, M. T., Goh, J. O., Chou, T. L., Chang, Y. L., & Tang, P. F. (2026). Midlife modulation of task switching brain activity reveals age specific neural adaptation. Scientific Reports, 16(1), Article 9735. https://doi.org/10.1038/s41598-026-39996-1 |
| Study 2015 | Richardson, D. R., Foxe, J. J., & Freedman, E. G. (2024). Reduced Proactive and Reactive Cognitive Flexibility in Older Adults Underlies Performance Costs During Dual-Task Walking: A Mobile Brain/Body Imaging (MoBI) Study. bioRxiv. https://doi.org/10.1101/2024.01.27.577090 |
| Study 2054 | Yeung, M. K. (2026). Task-switching and frontal cortex functioning across adulthood: An fNIRS study. NeuroImage (Orlando, Fla.), 310, Article 121160. https://doi.org/10.1016/j.neuroimage.2025.121160 |
| Study 2101 | Schils, L. A. P., Koch, I., Huang, P.-C., Hsieh, S., & Stephan, D. N. (2025). The Role of Task-Set Preparation and Modality Compatibility in Age-Related Effects on Cognitive Flexibility: Evidence From Task Switching. Psychology and Aging, 40(7), 765–777. https://doi.org/10.1037/pag0000907 |
| *Note*. Complete references are provided for all reviewed studies. Study numbers were assigned during the screening stage. Studies not presented in this table were excluded based on the inclusion and exclusion criteria of the systematic review. | |

**Content Relevant to Results**

**Supplementary Figure 1**

*Publication Year for the Records (N = 73) Included in the Systematic Review*

| **Supplementary Table 3**  *Summary of Base-Tasks* | | | |
| --- | --- | --- | --- |
| **Base-Tasks Category** | **Base-Tasks** | **Study Count** | **Year Range** |
| Digit Tasks | | | |
|  | Digit parity (odd/even), digit comparison (greater/less than 5) | 15 | 2006-2024 |
| Colour and Shape Tasks | | | |
|  | Shape discrimination (e.g., circle/square), colour discrimination (e.g., red/green) | 9 | 2010-2018 |
|  | Form discrimination (line either horizontal or vertical, cross), colour discrimination (cyan/magenta/white) | 1 | 2001 |
| Colour and Letter Tasks | | | |
|  | Colour (or luminance) discrimination (e.g., red/green/blue), letter discrimination (e.g., I/S/O) | 4 | 2008-2020 |
|  | Stroop Task in task-switching paradigm | 6 | 2005-2008 |
| Digit and Letter Tasks | | | |
|  | Digit characteristics (odd/ even), letter characteristics (consonant/ vowel) | 5 | 2008-2020 |
|  | Digit characteristics (greater/less than 5), letter characteristics (consonant/ vowel) | 4 | 2000-2024 |
| Switch Between Three Tasks | | | |
|  | Digit characteristics (odd/even), letter characteristics (uppercase/lowercase), and colour discrimination (red/blue) | 2 | 2015 |
|  | Shape discrimination (circle/square), colour discrimination (red/green), and form discrimination (plus vs line) | 1 | 2001 |
|  | Digit characteristics (greater/less than 5, odd/even, larger/smaller font) | 2 | 2011, 2015 |
|  | Shape characteristics (circle/square, red/blue, small/large) | 2 | 2012 |
| Spatial Tasks |  |  |  |
|  | Location of the stimulus (upper/lower, right/left) | 4 | 2001, 2025 |
|  | Eye position (look toward or away from stimuli) | 1 | 2004 |
|  | Visuospatial task (corresponding or opposite space-key mapping) | 1 | 2012 |
| Semantic and Non-Semantic Tasks | | | |
|  | Semantic discrimination (e.g., fruit/vegetable), size discrimination (large/small) | 4 | 2009-2017 |
|  | Semantic characteristic (animal/nonanimal), syllable characteristic (one/two syllables) | 1 | 2006 |
| Digit and Element Number Tasks | | | |
|  | Digit value (less than/more than 5), element number (whether array of numbers has more than or less than five elements, all same number) | 4 | 1999-2001 |
| Letter Tasks | | | |
|  | Letter characteristics (upper/lower, consonant/vowel) | 4 | 2014-2016 |
| Words Generation Tasks | | | |
|  | Participant generates exemplars based on given category (switch category) | 2 | 2000 |
| Task Unique to a Single Study | | | |
|  | Continuous calculation (average when numeric stimuli differed by two / addition or subtraction when numbers differed by one) | 1 | 2007 |
|  | Switch between different calculation tasks | 1 | 2025 |
|  | Shape discrimination (circle/diamond), size discrimination (small/large) | 1 | 2016 |
|  | Shape discriminations | 1 | 2024 |
|  | Letter discrimination (vowel/consonant), position discrimination (lower/upper) | 1 | 2012 |
|  | Digit discrimination (1/2) or colour discrimination (blue/yellow) | 1 | 2023 |
|  | Digit discrimination or size discrimination | 1 | 2026 |
|  | Switch between either shape-discrimination (same/different shape) or colour-discrimination (same/different colour) tasks, inner-stimulus or outer-stimulus, or double-switch | 1 | 2023 |
| Multiple Paradigms | | | |
|  | 1. Letter classification (A, E, I, U, C, G, K, Z) or digit characteristics (odd/even), informatively cued task  2. Letter classification or digit characteristics (odd/even), non-informatively cued task  3. Colour (Green, Orange, Light Blue) or shape classification task (Triangle, square, hexagon), informatively cued task | 1 | 2015 |
|  | 1. Digit characteristics (greater/less than 5), letter characteristics (consonant, vowel)  2. Digit characteristics (greater/less than 5, odd/even) | 1 | 2015 |
|  | 1. Space discrimination (left/right), word discrimination (left/right)  2. Space discrimination (matching the direction of arrow, opposite to the arrow) | 1 | 2012 |
|  | 1. Linguistic (presented picture, then verbally name the object in Catalan/Spanish)  2. Non-linguistic (shape discrimination: square/circle/triangle, Colour discrimination: green/blue/unspecified) | 1 | 2013 |
| *Note*. Study Count: Number of studies used the listed base tasks. For more details, please see Paradigm Information Spreadsheet in supplementary materials. | | | |

| **Supplementary Table 4**  *Effect Sizes of Local-Switch Costs (Reaction Time): Descriptive Summary* | | | | | |
| --- | --- | --- | --- | --- | --- |
| **Grouped by** | ***N*** | ***Min.*** | ***Max.*** | ***Mean*** | ***Median*** |
| **Base-Tasks** |  |  |  |  |  |
| Colour/Digit | 1 | 0.41 | 0.41 | 0.41 | - |
| Colour/Letter | 6 | 0.11 | 0.53 | 0.27 | 0.24 |
| Colour/Shape | 5 | 0.16 | 0.80 | 0.62 | 0.74 |
| Digit/Element | 1 | 0.47 | 0.47 | 0.47 | - |
| Digit/Letter | 3 | 0.16 | 0.80 | 0.42 | 0.29 |
| Digits | 6 | 0.26 | 0.97 | 0.73 | 0.80 |
| Others | 2 | 0.29 | 0.70 | - | - |
| Spatial | 3 | 0.62 | 0.74 | 0.70 | 0.74 |
| Three Tasks | 3 | 0.48 | 0.67 | 0.55 | 0.49 |
| **Switch-Rules** |  |  |  |  |  |
| Colour | 7 | 0.16 | 0.80 | 0.41 | 0.29 |
| Memorise | 1 | 0.48 | 0.48 | 0.48 | - |
| Modality | 1 | 0.62 | 0.62 | 0.62 | - |
| Double | 6 | 0.49 | 0.97 | 0.77 | 0.80 |
| Unspecified | 1 | 0.41 | 0.41 | 0.41 | - |
| Verbal | 1 | 0.67 | 0.67 | 0.67 | - |
| Visual | 4 | 0.29 | 0.74 | 0.63 | 0.74 |
| Words | 9 | 0.11 | 0.80 | 0.43 | 0.29 |
| **All Studies** | 30 | 0.11 | 0.97 | 0.53 | 0.58 |
| *Note.* *N* = Number of reported ANOVA test results. The number of studies in each category is unevenly distributed. The interpretation of categories with less studies should be considered with extra caution, as smaller studies numbers made their results less generalisable to the represented group. | | | | | |

| **Supplementary Table 5**  *Effect Sizes of Local-Switch Costs (Accuracy): Descriptive Summary* | | | | | |
| --- | --- | --- | --- | --- | --- |
|  | ***N*** | ***Min.*** | ***Max.*** | ***Mean*** | ***Median*** |
| **Base-Tasks** |  |  |  |  |  |
| Colour/Digit | 1 | 0.35 | 0.35 | 0.35 | - |
| Colour/Letter | 3 | 0.15 | 0.27 | 0.22 | 0.23 |
| Colour/Shape | 2 | 0.16 | 0.37 | 0.25 | 0.25 |
| Digits | 3 | 0.13 | 0.57 | 0.40 | 0.49 |
| Others | 1 | 0.07 | 0.07 | 0.07 | - |
| Semantic | 1 | 0.54 | 0.54 | 0.54 | - |
| Spatial | 3 | 0.23 | 0.48 | 0.36 | 0.35 |
| Three Tasks | 2 | 0.16 | 0.50 | 0.33 | 0.33 |
| **Switch-Rules** |  |  |  |  |  |
| Colour | 1 | 0.07 | 0.07 | 0.07 | - |
| Double | 4 | 0.13 | 0.57 | 0.34 | 0.32 |
| Memorise | 1 | 0.50 | 0.50 | 0.50 | - |
| Modality | 1 | 0.48 | 0.48 | 0.48 | - |
| Unspecified | 1 | 0.35 | 0.35 | 0.35 | - |
| Visual | 3 | 0.23 | 0.35 | 0.27 | 0.23 |
| Words | 5 | 0.15 | 0.54 | 0.29 | 0.27 |
| **All studies** | 16 | 0.07 | 0.57 | 0.31 | 0.30 |
| Notes. *N* = Number of reported ANOVA test results. The number of studies in each category is unevenly distributed. The interpretation of categories with less studies should be considered with extra caution, as smaller studies numbers made their results less generalisable to the represented group. | | | | | |

| **Supplementary Table 6**  *Effect Sizes of Global-Switch Costs (Reaction Time): Descriptive Summary* | | | | | |
| --- | --- | --- | --- | --- | --- |
| **Grouped by** | ***N*** | ***Min.*** | ***Max.*** | ***Mean*** | ***Median*** |
| **Base-Tasks** |  |  |  |  |  |
| Colour/Letter | 2 | 0.81 | 0.84 | 0.82 | 0.82 |
| Colour/Shape | 2 | 0.76 | 0.77 | 0.76 | 0.76 |
| Digit/Element | 1 | 0.83 | 0.83 | 0.83 | - |
| Digit/Letter | 4 | 0.29 | 0.71 | 0.45 | 0.39 |
| Digits | 4 | 0.67 | 0.93 | 0.81 | 0.81 |
| Letter/Position | 1 | 0.80 | 0.80 | 0.80 | - |
| Letters | 2 | 0.85 | 0.87 | 0.86 | 0.86 |
| Others | 1 | 0.23 | 0.23 | 0.23 | - |
| Spatial | 1 | 0.64 | 0.64 | 0.64 | - |
| Three Tasks | 2 | 0.83 | 0.88 | 0.85 | 0.85 |
| **Switch-Rules** |  |  |  |  |  |
| Colour | 8 | 0.29 | 0.87 | 0.70 | 0.81 |
| Double | 2 | 0.81 | 0.93 | 0.87 | 0.87 |
| Memorise | 1 | 0.83 | 0.83 | 0.83 | - |
| Modality | 1 | 0.64 | 0.64 | 0.64 | - |
| Unspecified | 1 | 0.63 | 0.63 | 0.63 | - |
| Visual | 2 | 0.23 | 0.77 | 0.50 | 0.50 |
| Words | 6 | 0.31 | 0.88 | 0.72 | 0.79 |
| **All Studies** | 20 | 0.29 | 0.93 | 0.71 | 0.81 |
| *Note.* *N* = Number of reported ANOVA test results. The number of studies in each categories is unevenly distributed. The interpretation of categories with less studies should be considered with extra caution, as smaller studies numbers made their results less generalisable to the represented group. | | | | | |

| **Supplementary Table 7**  *Effect Sizes of Global-Switch Costs (Accuracy): Descriptive Summary* | | | | | |
| --- | --- | --- | --- | --- | --- |
| **Grouped by** | ***N*** | ***Min.*** | ***Max.*** | ***Mean*** | ***Median*** |
| **Base-Tasks** |  |  |  |  |  |
| Colour/Digit | 1 | 0.17 | 0.17 | 0.17 | - |
| Colour/Letter | 1 | 0.52 | 0.52 | 0.52 | - |
| Colour/Shape | 1 | 0.29 | 0.29 | 0.29 | - |
| Digit/Element | 1 | 0.42 | 0.42 | 0.42 | - |
| Digit/Letter | 2 | 0.16 | 0.38 | 0.27 | 0.27 |
| Digits | 2 | 0.25 | 0.44 | 0.34 | 0.34 |
| Letter/Position | 1 | 0.43 | 0.43 | 0.43 | - |
| Letters | 2 | 0.40 | 0.54 | 0.47 | 0.47 |
| Spatial | 1 | 0.48 | 0.48 | 0.48 | - |
| Three Tasks | 2 | 0.30 | 0.46 | 0.38 | 0.38 |
| **Switch-Rules** |  |  |  |  |  |
| Colour | 6 | 0.16 | 0.54 | 0.40 | 0.43 |
| Double | 1 | 0.25 | 0.25 | 0.25 | - |
| Memorise | 1 | 0.46 | 0.46 | 0.46 | - |
| Modality | 1 | 0.48 | 0.48 | 0.48 | - |
| Unspecified | 1 | 0.17 | 0.17 | 0.17 | - |
| Visual | 1 | 0.52 | 0.52 | 0.52 | - |
| Words | 3 | 0.29 | 0.38 | 0.33 | 0.30 |
| **All Studies** | 14 | 0.16 | 0.54 | 0.38 | 0.41 |
| *Note.* *N* = Number of reported ANOVA test results. The number of studies in each category is unevenly distributed. The interpretation of categories with less studies should be considered with extra caution, as smaller studies’ results are less generalisable to the represented group. | | | | | |

**Supplementary Figure 2**

*Effect Sizes of Local-Switch Costs (Reaction Time)*

*Note.* This figure depicts effect sizes by study, grouped by base-task categories (colour) and switch-rule categories (shape). The red line indicates the mean of the effect sizes. The blue lines indicate the minimum and maximum effect sizes. As special cases, the study with Colour/Digit base-task and Unspecified switch-rule (partial eta-squared = 0.41), and the study with Spatial base-task and Modality switch-rule (partial eta-squared = 0.62) were excluded from the figure to increase figure clarity.

**Supplementary Figure 3**

*Effect Sizes of Local-Switch Costs (Accuracy)*

*Note.* This figure depicts effect sizes by study, grouped by base-task categories (colour) and switch-rule categories (shape). The red line indicates the mean of the effect sizes. The blue lines indicate the minimum and maximum effect sizes. As special cases, the study with Colour/Digit base-task and Unspecified switch-rule (partial eta-squared = 0.35), and the study with Spatial base-task and Modality switch-rule (partial eta-squared = 0.48) were excluded from the figure to increase figure clarity.

**Supplementary Figure 4**

*Effect Sizes of Global-Switch Costs (Reaction Time)*

*Note.* This figure depicts effect sizes by study, grouped by base-task categories (colour) and switch-rule categories (shape). The red line indicates the mean of the effect sizes. The blue lines indicate the minimum and maximum effect sizes. As special cases, the study with Spatial base-task and Modality switch-rule (partial eta-squared = 0.64) were excluded from the figure to increase figure clarity.

**Supplementary Figure 5**

*Effect Sizes of Global-Switch Costs (Accuracy)*

*Note.* This figure depicts effect sizes by study, grouped by base-task categories (colour) and switch-rule categories (shape). The red line indicates the mean of the effect sizes. The blue lines indicate the minimum and maximum effect sizes. As special cases, the study with Colour/Digit base-task and Unspecified switch-rule (partial eta-squared = 0.17), and the study with Spatial base-task and Modality switch-rule (partial eta-squared = 0.48) were excluded from the figure to increase figure clarity.

**Supplementary Materials: Code for Calculating Partial Eta-Squared**

**R Code:**

```
## Define the Function for Partial Eta-Squared

partial_eta_approximator <- function(F_stat, df_num, df_denom){
(F_stat*df_num) / ((F_stat * df_num) + df_denom)
}


## An Example For Calculating Local-Switch Costs Measured by RT

Dataset$eta_square_Approx <- NA

for(i in 1:nrow(Dataset)){
Dataset$eta_square_Approx[i] <- partial_eta_approximator(Dataset$F_stats[i], Dataset$df1[i], Dataset$df2[i])
}
```

**Supplementary Materials: Descriptive Assessment of the Combinations of Base-Tasks and Switch-Rules**

**Among the 15 studies using digit base-tasks, the switch-rules included:**

- colour cues (5 studies)
- memorisation (1 study)
- two switch-rules or combined switch-rule methods (5 studies)
- unspecified switch-rules (1 study)
- visual cues (2 studies)
- word cues (1 study).

**Among the 26 studies using word-cued switch-rules, the base-tasks included:**

- colour + letter (3 studies)
- colour + shape (6 studies)
- digit + letter (2 studies)
- digits (1 study)
- digits + element number (2 studies)
- face categorisation (1 study)
- semantic + size tasks (2 studies)
- semantic + syllable tasks (1 study)
- shape + size tasks (1 study)
- spatial tasks (1 study)
- Stroop tasks (3 studies)
- three-task paradigms (3 studies).
